# When CuCl_4_^2–^ and CuBr_4_^2–^ Form Anion···Anion Networks Assembled via Cu···Cl/Br Regium Bonds

**DOI:** 10.1021/acs.cgd.5c00238

**Published:** 2025-06-05

**Authors:** Cristina Lo Iacono, Andrea Pizzi, Kamran T. Mahmudov, Rosa M. Gomila, Antonio Frontera, Giuseppe Resnati

**Affiliations:** † NFMLab, Department Chemistry Materials, Chemical Engineering “Giulio Natta”, 18981 Politecnico di Milano, Milano 20133, Italy; ‡ Excellence Center, Baku State University, Baku AZ 1148, Azerbaijan; § Western Caspian University, Baku AZ 1001, Azerbaijan; ∥ Department of Chemistry, Universitat de les Illes Balears, Crta. de Valldemossa, Palma de Mallorca, Balears 07122, Spain

## Abstract

This study presents the design, preparation, and single-crystal X-ray characterization of CuCl_4_^2–^ and CuBr_4_^2–^ salts, wherein dianions self-assemble into supramolecular (4,4) networks via short Cu···Cl/Br contacts (regium bonds). In the described salts, the organic cations play a pivotal role in promoting the ability of Cu atoms to act as electrophiles. The used cations are the protonated forms of primary amines and form a tight network of N^+^H···Cl/Br hydrogen bonds that promote the regium bond donor ability of Cu by dissipating the anion negative charge. Calculations, namely determination of the molecular electrostatic potential, quantum theory of atoms in molecules and natural bond orbital analyses and other approaches, afford a comprehensive understanding of the Cu···Cl/Br short contacts identified via crystallography and confirm the attractive character of the charge transfer from an occupied lone pair on the halogen of one anion to an empty σ* orbital on copper of another anion.

## Graphical abstract

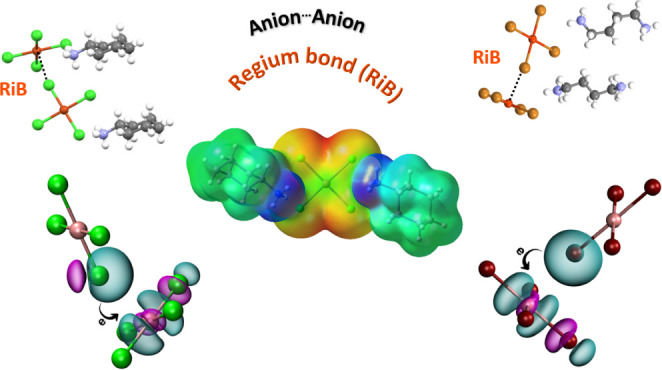

## Introduction

Noncovalent interactions play a fundamental role in determining the properties of molecular systems.^1^ Interactions drive the self-assembly of molecules into nano-, micro-, and macro-structures, and their relevance extends to several fields, from pharmacology to materials science.^2–4^ A fairly useful and general feature for understanding interaction formation is the anisotropic electron density distribution at atoms’ surface;^5^ in fact, the surface of covalently bonded atoms typically shows the presence of regions of depleted electron density as well as regions of excess electron density. The former regions commonly called σ- ^6,7^ and π-holes^8,9^ are frequently characterized by a positive electrostatic potential and act as electrophilic sites that can engage in attractive interactions with atoms/regions of the same or other molecules featuring negative electrostatic potential. This behavior, originally observed in p-block elements,^10–14^ has since been extended to a wide range of elements of d-blocks.^15–20^ Over the past decade, this understanding has been applied to the group 11 elements, i.e., copper (Cu), silver (Ag), and gold (Au). These elements, in some of their compounds, possess regions of strong electrophilic character, just due to the anisotropic electron density at atoms’ periphery. The interactions between these metals and nucleophiles are often named regium bonds (RiBs)^21^ or coinage bonds (CiBs),^22,23^ with these terms highlighting a unique subset of the noncovalent interactions formed by group 11 metals.^24,25^

In particular, it has been demonstrated that sometimes polyatomic anions behave not only as σ- and π-hole bond acceptors (nucleophiles), as expected for anions, but also as σ- and π-hole bond donors (electrophiles), and resulting anion···anion interactions form 1D, 2D, or 3D supramolecular assemblies.^26^ Also for these unexpected supramolecular adducts, the electrophilic atom of polyatomic anions acting as σ-/π-hole bond donor can be an element of both the p- and d-blocks (e.g., iodine or selenium^27–29^ and manganese or rhenium,^30,31^ respectively). As to group 11 elements, regium bonds involving gold tetrahalides (e.g., AuCl_4_^–^) or tetracyanides (Au­(CN)_4_^–^) have been well-studied, and the obtained results have promoted scientific interest in the role of metal-containing anions in advanced materials development.^32–35^ Analogous features in the derivatives of the lighter group 11 elements have not been studied. Indeed, the exploitation of the regium bond in the self-assembly of copper-based anions such as CuCl_4_^2–^ and CuBr_4_^2–^ (Scheme 1) has not been explored. Here, the main challenge lies in the significant Coulombic repulsion between these anions. In fact, it may be expected that their involvement in the formation of attractive anion···anion interactions is more challenging than for AuCl_4_^–^ as the net negative charge of the copper­(II) tetrachlorides is two times that of Au­(III) tetrachlorides.

**Scheme 1:**
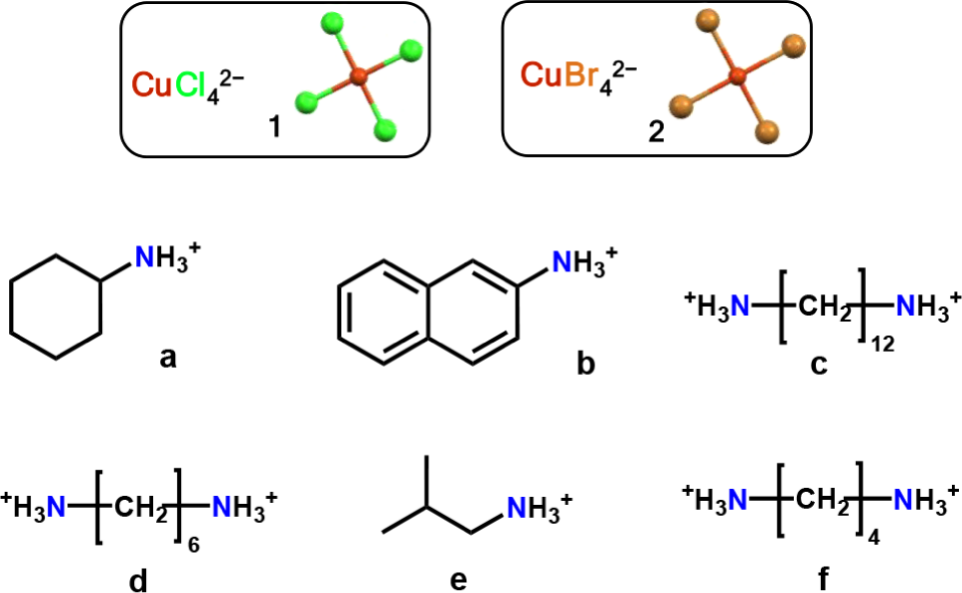
Structural Formulas of Cations and Anions of the Terachlorido-cuprates­(II) and Tetrabromido Analogues Studied in this Paper

However, recent theoretical and experimental studies suggest that under certain conditions, Coulombic repulsion can be overcome and short contacts can be formed even in the case of dianions.^36–39^ In this study, we present the first case where the self-assembly of CuCl_4_^2–^ or CuBr_4_^2–^ dianions is designed and the obtained anion···anion interactions are rationalized as regium bonds. In detail, a series of hybrid organic–inorganic tetrachlorido- and tetrabromidocuprate­(II) salts have been synthesized using primary aliphatic or aromatic amines as sources of the cationic component (Scheme 1). Single-crystal X-ray analyses show that anions adopt a distorted square planar arrangement and assemble into (4,4) networks via short Cl—Cu···Cl—Cu regium bonds. Detailed theoretical studies of these contacts prove that these anion···anion interactions are attractive, albeit quite weakly attractive.

## Experimental Section

### Materials and Methods

Starting materials and solvents were purchased from Merck and abcr and used without further purification for synthesis and crystallization.

IR spectra were obtained using a Nicolet Nexus FT-IR spectrometer equipped with a UATR unit.

^1^H and^13^C NMR spectra were recorded at ambient temperature on a Bruker AV-400 spectrometer by using DMSO-*d6* as the solvent. All chemical shifts in Supporting Information are given in ppm.

Preparation of adducts **1a**–**d**: A methanol solution of CuCl_2_ was added to aqueous HCl (6 N, 6 equiv); the resulting mixture was added under stirring to an equimolar amount of the amine (cyclohexylamine for preparing **1a**, 2-naphthylamine for preparing **2b**, 1,12-diaminododecane for preparing **1c**, and 1,6-diaminohexane for preparing **1d**). Adducts **2a,e,f** were prepared via the same procedure and by using CuBr_2_, aqueous HBr, and cyclohexylamine for **2a**, isobutylamine for **2e**, and 1,4-diaminobutane for **2f**. The mixtures were left at room temperature in a clear borosilicate vial. Crystals suitable for X-ray diffraction were formed through slow isothermal evaporation of solvents.

The single-crystal data of **1a**–**d** and **2a,e,f** were collected at room temperature or 100 K using an XtaLAB Synergy diffractometer equipped with a HyPix detector. Unit cell refinement and data reduction were performed using CrysAlisPro 1.171.41.98a. Structures were solved by direct methods using SHELXT^40,41^ and refined by full-matrix least-squares on F^2^ with anisotropic displacement parameters for the non-H atoms using Olex2.^42^ Absorption correction was performed based on a multiscan procedure. Figures were prepared using Mercury.^43^ CCDC deposition numbers 2421519, 2421520, 2421577, 2421578, 2421579, 2422984 and 2421582 contain the supplementary crystallographic data for this paper. These data are provided free of charge by the Cambridge Crystallographic Data Centre.

The geometries of the complexes included in this study were computed at the PBE0-D3/def2-TZVP level of theory^44–46^ using the crystallographic coordinates within the Turbomole 7.7 program.^47^ The “atoms-in-molecules” (AIM)^48^ analysis of the electron density was performed at the same level of theory using the Multiwfn program.^49^ The reduced density gradient (RDG) 2D plots^50^ and ELF^51^ 2D plots were computed using the Multiwfn program.^49^ The QTAIM analysis was represented using the VMD software.^52^ The Laplacian of the electron density can be decomposed into the sum of contributions along the three principal axes of maximal variation, giving the three eigenvalues of the Hessian matrix (λ_1_, λ_2_, and λ_3_). The sign of λ_2_ can be utilized to distinguish bonding (attractive, λ_2_ < 0) weak interactions from nonbonding ones (repulsive, λ_2_ > 0).^50^ Natural bond orbital (NBO)^53^ calculations were performed using the NBO7.0 program.^54^

The molecular electrostatic potential (MEP) surface plots were generated using the GaussView 6.0 program^55^ at two different isodensity values: 0.002 au, which encloses approximately 98% of the total electron density, and 0.0044 au, which includes about 97%. The latter was employed to evaluate how MEP values vary with a reduction in surface size, which is particularly relevant given that the RiB distances discussed herein are shorter than the sum of the van der Waals radii (vide infra). To determine the percentage of electron density enclosed at each isovalue, the Spartan software^56^ was used to numerically integrate the electron density in the space outside the isosurface, thereby quantifying the excluded fraction and its complement within the surface.

## Results and Discussion

### Cambridge Structural Database (CSD) Analyses

In the CSD (Conquest version 2024.1.0), the numbers of structures containing isolated CuCl_4_^2–^ or CuBr_4_^2–^ units are large enough to allow for formulating a robust working hypothesis. Both anions adopt the whole assortment of possible geometries, spanning the tetrahedral, seesaw, or square planar arrangements and the corresponding distorted ones (see SI). Searches for short Cu···Cl/Br contacts between two anions reveal that these interactions are uncommon for CuCl_4_^2–^ anions (13% ca.) and very uncommon for CuBr_4_^2–^anions (2% ca). Interestingly, in the very large majority of the structures wherein these contacts are present, the cations are the protonated form of primary amines or poly-nitrogen organic compounds (e.g., polysecondary amines, guanidinium derivatives).

We reasoned that this feature may play a particularly important role in enabling the formation of short Cu···Cl/Br contacts between anions. The protonated form of primary amines and poly-nitrogen organic compounds is particularly tailored to form numerous and strong hydrogen bonds (HBs) with CuCl_4_^2–^ and CuBr_4_^2–^. These HBs dissipate the anion’s negative charge, namely, promote the regium bond donor ability of copper by influencing its electrostatic potential. The relevance of these HBs in favoring the formation of anion···anion regium bonds is confirmed by the fact that no such interactions are observed in any of the nearly 60 salts of CuCl_4_^2–^ and CuBr_4_^2–^ where the cations are quaternary ammonium species (e.g., (Et_4_N)_2_CuCl_4_,^57^ 1,4-dimethyl-1,4-diazoniabicyclo(2.2.2)­octane tetrachlorido-copper­(II),^58^ bis­(trimethylphenylammonium) tetrabromido-copper­(II),^59^ and (Et_2_Me_2_N)_2_CuBr_4_).^60^

Moving from the working hypothesis described above, we prepared the tetrachlorido- and tetrabromido-cuprate­(II) salts **1a**–**d** and **2a,e,f** (Scheme 1) and determined the details of their structures in the solid via single-crystal X-ray analyses.

### Single-Crystal X-ray Analysis of Tetrachlorido- and Tetrabromido-Cuprates­(II) 1a–d, 2a,e,f

The strongest attractive force between the different units in the examined salts is probably the cation–anion Coulombic attraction. A tight network of H···Cl/Br HBs is present in all salts, and these contacts can be as short as 234.5 and 237.4 pm in **1b** and **1c** or 250.3 and 245.3 pm in **2e** and **2f**. The presence of this network favors, or is favored by, the segregation of cations and anions into alternating layers glued at their interfaces by the HBs. This layered arrangement, present for all examined salts, is exemplified in Figure 1 for **1a** and **2e** (see Figures S1–S3 for other salts). Importantly, the HBs help to decrease the negative charge of the CuCl_4_^2–^ and CuBr_4_^2–^ anions, which become more efficient acceptors of electron density.

**Figure 1:**
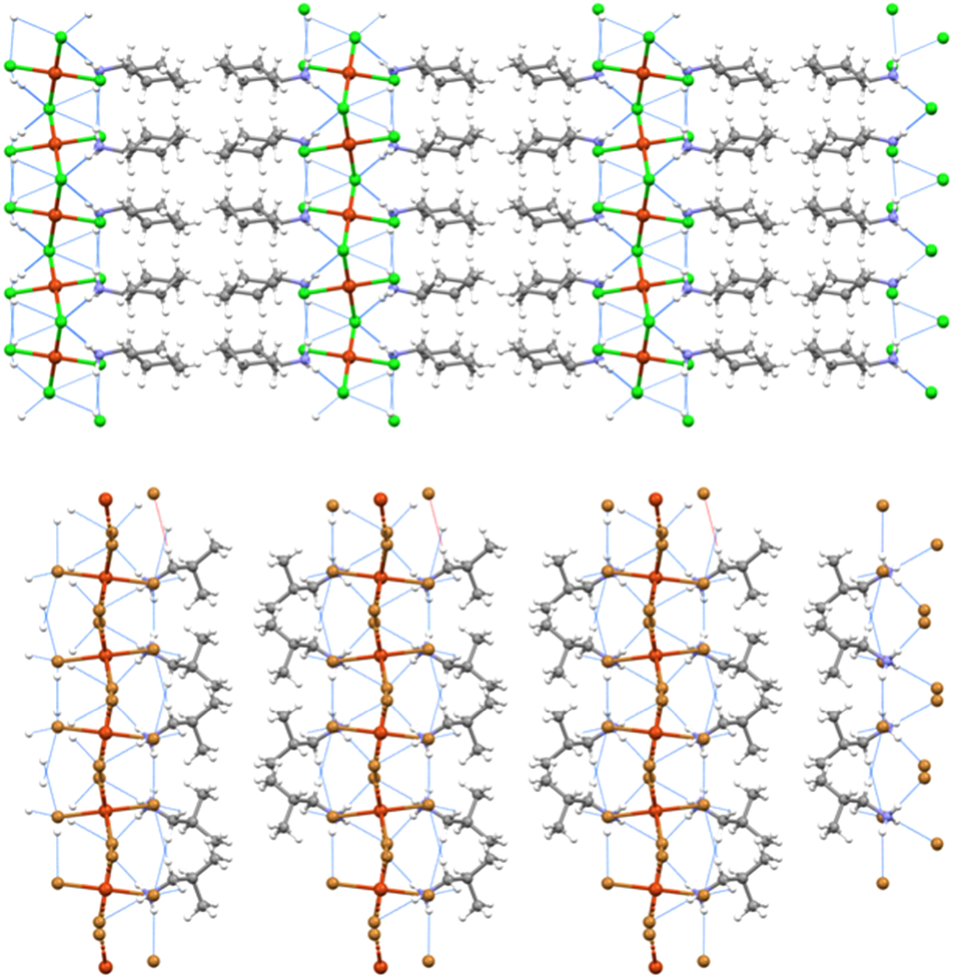
Partial view of the crystal packing (ball and stick representation) of the tetrachlorido salt **1a** (along the *b* axis, top) and the tetrabromido salt **2e** (along the *c* axis, bottom) evidencing the layered architecture, the HBs (blue lines) at the layers interfaces, and the opposite orientation of cations in the organic layer, resulting into an ABBA patter. Color code: whitish, hydrogen; gray, carbon; indigo, nitrogen; orange, copper; bright green, chlorine; light brown, bromine.

The copper atoms act as bidentate RiB donors (electrophilic atoms), and two different halogens of any dianion act as monodentate RiB acceptors (nucleophilic atoms). CuCl_4_^2–^ and CuBr_4_^2–^ dianions function as self-complementary and tetradentate tectons, and fairly flat (4,4) networks are formed wherein anions sit at the nodes. The supramolecular anion···anion networks formed by the Cu···Cl/Br regium bonds in **1b** and **2a** are reported in Figure 2 (see Figures S4,S5 for other salts). The dianions adopt a square planar conformation, with copper atoms being approached by two halogens of two different and adjacent anions from the two sides of the anion plane and after directions orthogonal to the plane.

**Figure 2:**
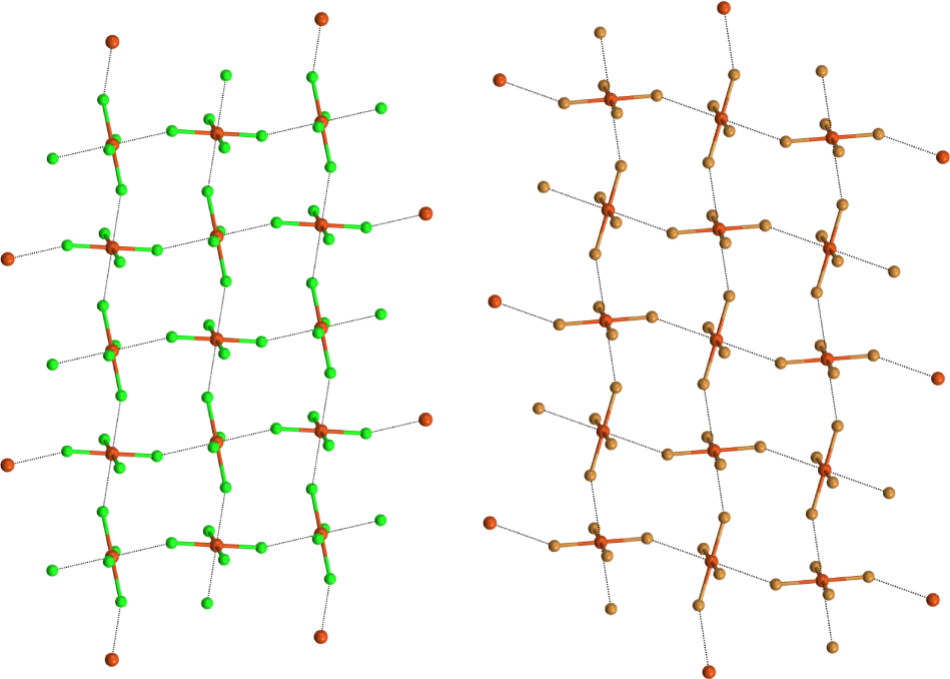
Partial view of the regium bonded (4,4) network (ball and stick representation) in tetrachlorido salt **1b** (left) and tetrabromido salt **2a** (right). RiBs are black lines. Color code: orange, copper; bright green, chlorine; light brown, bromine.

Specifically, in salt **1a**, each ammonium group of the cyclohexylammonium cation forms hydrogen bonds with three Cl atoms of different CuCl_4_^2–^ units (Figure 3). The CuCl_4_^2–^ anions adopt a square planar geometry (Cl—Cu—Cl angles are 88.83° and 91.17°), and two Cl atoms of two adjacent anions approach Cu atoms from opposite sides and are almost orthogonal to the plane of the anion. Cu···Cl contacts are 342.1 pm long (Nc = 0.90),^61^ and Cl—Cu···Cl angles span the range 84.18°–95.82°. These interactions form infinite (4,4) networks wherein CuCl_4_^2–^ units are connected to each other through four regium bonds (Figure S4). The inorganic layers of supramolecular anion networks alternate with organic layers formed by noninteracting, oppositely oriented cyclohexylammonium cations (ABBA pattern, A = CuCl_4_^2–^, B = organic cation) (Figure 1). The N^+^H_3_ groups are localized close to the inorganic layers and form a network of H···Cl HBs that bind the organic layer to the two adjacent inorganic layers.

**Figure 3:**
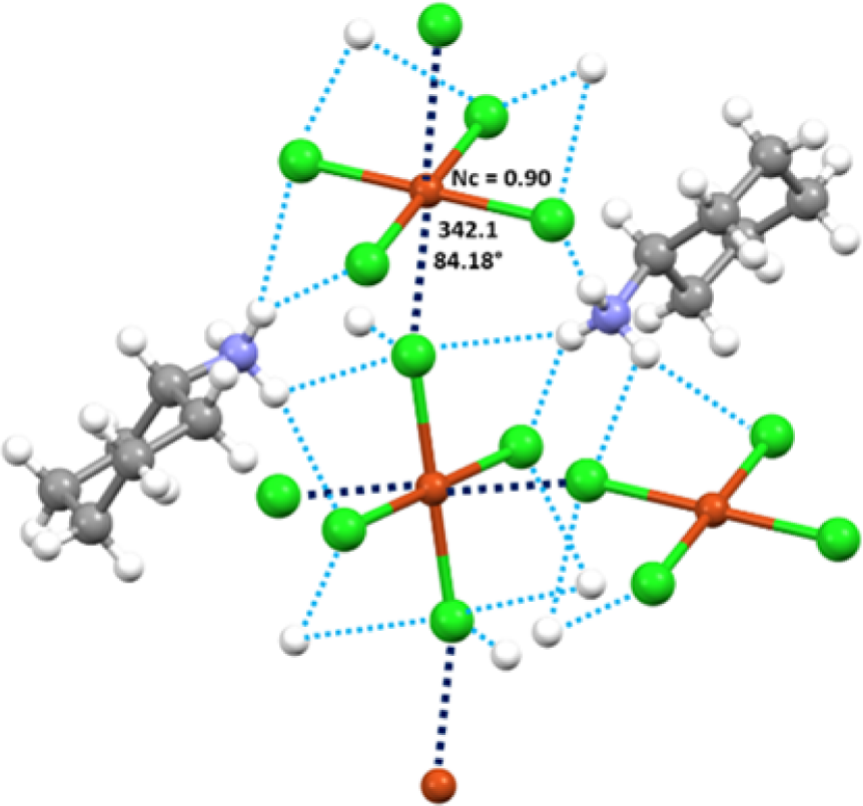
Ball and stick representation of the interaction pattern in **1a**. The RiBs are black dashed lines, and HBs are light blue dashed lines. The angles, lengths, and Nc of the RiBs are shown near the contact. Color code: whitish, hydrogen; gray, carbon; indigo, nitrogen; orange, copper; bright green, chlorine.

A similar ABBA interaction pattern (A = CuCl_4_^2–^, B = organic cation) is observed in salt **1b** (Figure S1), where the 2-naphthylammonium cations form N^+^H_3_···Cl hydrogen bonds with three different CuCl_4_^2–^ units and a CH···Cl bond with a fourth CuCl_4_^2–^ unit. Similar to salt **1a**, the square planar CuCl_4_^2–^ anions self-assemble into regium-bonded (4,4) networks (Figure 2) via quite short Cu···Cl contacts (308.2 pm, Nc = 0.81) and Cl–Cu···Cl angles that are even closer to orthogonality than in **1a** (they vary between 88.84° and 91.07°).

In salts **1c** and **1d**, cations are linear α,ω-diammonium alkanes and alternate with anions adopting an ABA pattern (A = CuCl_4_^2–^, B= linear α,ω-diammonium alkanes) (Figures 4 and S2). The anion···anion networks formed by CuCl_4_^2–^ are quite similar to those in **1a**,**b** (Cu···Cl separations are 298.3 and 299.8 pm (Nc = 0.79) in **1c** and 298.2 and 297.7 pm (Nc = 0.79 and 0.78) in **1d**; Cl—Cu···Cl angles vary in the range 85.81–94.19° and 85.03–9.97°, respectively) (Figure S5).

**Figure 4:**
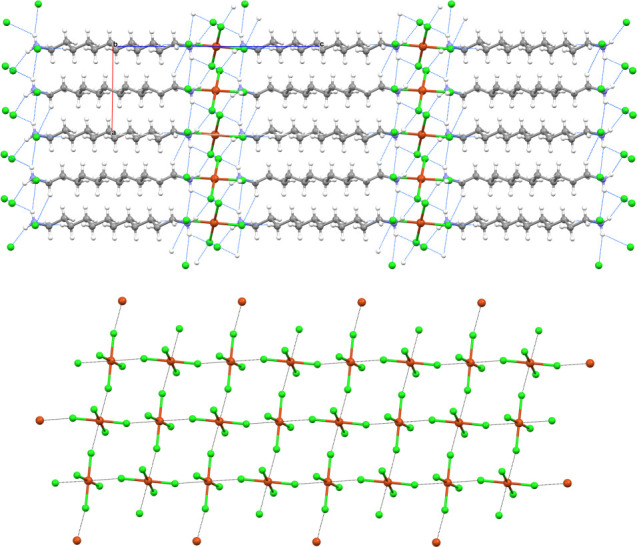
Partial view (ball and stick representation) of the crystal packing of the salt **1c** evidencing the alternating cation and anion layers adopting an ABA pattern; HBs are blue lines (top); the regium bonded network, RiBs are black lines (bottom). Color code: whitish, hydrogen; gray, carbon; indigo, nitrogen; orange, copper; bright green, chlorine.

The overall crystal packings of the CuBr_4_^2–^ salts **2a**, **2e,** and **2f** are very similar to those of CuCl_4_^2–^ salts **1a**-**d**. They consist of cationic and organic layers, which alternate with anionic and inorganic layers. In these layers, cations and anions adopt an ABBA pattern in **2a** and **2e** (A = CuBr_4_^2–^, B= protonated cyclohexyl and isobutyl amines, Figures 1 and S3) and an ABA pattern in **2f** (A = CuBr_4_^2–^, B = diprotonated 1,4-diaminobutane, Figure 5). The cationic and anionic layers are connected via N^+^H_3_···Br HBs, which dissipate the negative charge of CuBr_4_^2–^ anions and promote the ability of Cu atoms to establish Cu···Br contacts with adjacent CuBr_4_^2–^ anions and form (4,4) nets (Figure 2). Cu···Br separations are in the range 310.4–360.4 pm (Nc values are between 0.82 and 0.95), and Br—Cu···Br angles vary between 84.38 and 95.62°.

**Figure 5:**
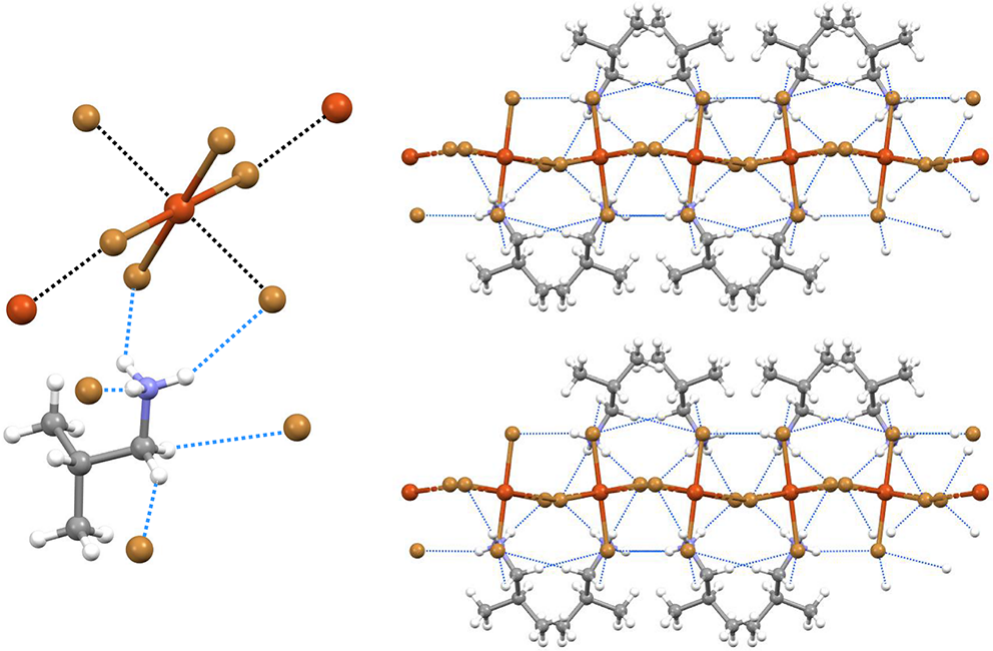
Ball and stick representation of the unit cell of the salt **2f**; HBs and RiBs are blue and black dotted lines (left); the layered structure with the ABBA pattern of **2f**, HBs are blue lines (right). Color code: whitish, hydrogen; gray, carbon; indigo, nitrogen; orange, copper; light brown, bromine.

### Theoretical Analyses of Salts 1a–d, 2a,e,f

The molecular electrostatic potential (MEP) surfaces of the CuCl_4_^2–^ and CuBr_4_^2–^ anions were computed to evaluate the anisotropic distribution of electron density in these species. The results, shown in Figure 6, confirm the anisotropic nature of these molecules and reveal that the MEP maximum is located at the Cu atom, with values of −166.9 and −158.8 kcal/mol for CuCl_4_^2–^ and CuBr_4_^2–^, respectively. The MEP minima are positioned along the bisector of the X—Cu—X angle (X = Cl, Br) in the molecular plane, with values of −220.3 and −202.1 kcal/mol for CuCl_4_^2–^ and CuBr_4_^2–^, respectively. Additionally, a local MEP maximum is observed along the Cu—X bond, further highlighting the anisotropy of the electrostatic potential distribution.

**Figure 6:**
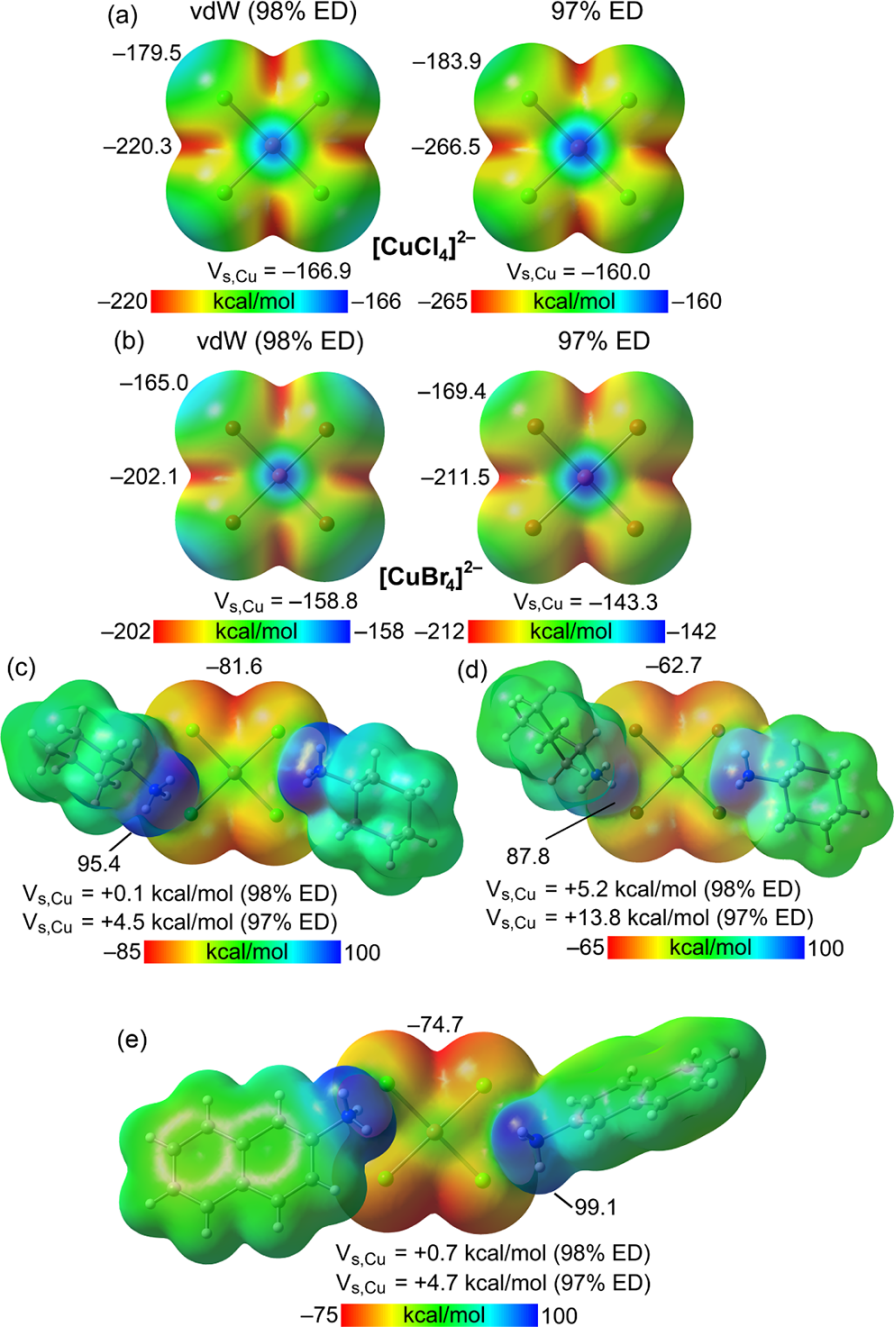
(a) MEP surfaces of [CuCl_4_]^2–^. Left: ED = 98%, isovalue 0.002 au; right ED = 97% (0.0044 au). (b) MEP surfaces of [CuBr_4_]^2–^. Left: ED = 98%, isovalue 0.002 au; right ED = 97%. (c) MEP surface of **1a**. (d) MEP surface of **2a**. (e) MEP surface of **1b**. In (c)–(e), the energies correspond to the 0.002 au isosurface apart from Cu, where the values using the ED = 97% surface (0.0044 au) are indicated. Energies are given in kcal/mol.

Given that the experimental normalized contacts (Ncs) for the Cu···X contacts are less than one, the MEP was recalculated using a surface that captures 97% (0.0044 au) of the electron density (ED) instead of 98% (0.002 au); see Section S4. This adjustment led to more positive MEP values at the Cu atom, specifically −160 kcal/mol and −143.3 kcal/mol for CuCl_4_^2–^ and CuBr_4_^2–^, respectively, namely, the MEP remains negative across the entire surface also on this isosurface.

To assess the influence of cationic counterions on the anion electron density, the MEP values at the Cu atom were recalculated, considering salts **1a**, **1b**, and **2a**. As illustrated in Figure 6c–e, the MEP maxima shift to the −N^+^H_3_ groups, ranging from 87.8 to 99.1 kcal/mol, while the MEP minima remain at the X—Cu—X bisectors, with values ranging from −62.7 to −81.6 kcal/mol. Notably, when counterions are present and the vdW surface is used, the MEP values at the Cu atom become slightly positive, with a further increase when the 97% ED surface is applied (up to 13.8 kcal/mol for **2a**). This cation effect of the anion electron density revealed by computational analyses is consistent with the dissipation of the anion charge allowed by the N^+^H_3_···Cl/Br HBs surmised by crystal structures analyses. Moreover, the electrostatic potential change suggests that the directional Cu···X contacts observed in the solid-state structures of these salts can be dependably interpreted as noncovalent regium bonds, where the Cu atom acts as an electrophilic site despite the dianionic nature of the CuX_4_^2–^ units.

Next, Quantum Theory of Atoms in Molecules (QTAIM) analysis was employed to investigate the anion···anion dimers in **1a**, **1b**, and **2a**, in both the absence and presence of counterions, as shown in Figure 7. In these dimers, the anions are linked by a bond critical point (BCP) (represented as a magenta sphere) and a bond path (depicted as a solid orange line) connecting the Cu atom to the X atom, thereby confirming the presence of Cu···X contacts.

**Figure 7:**
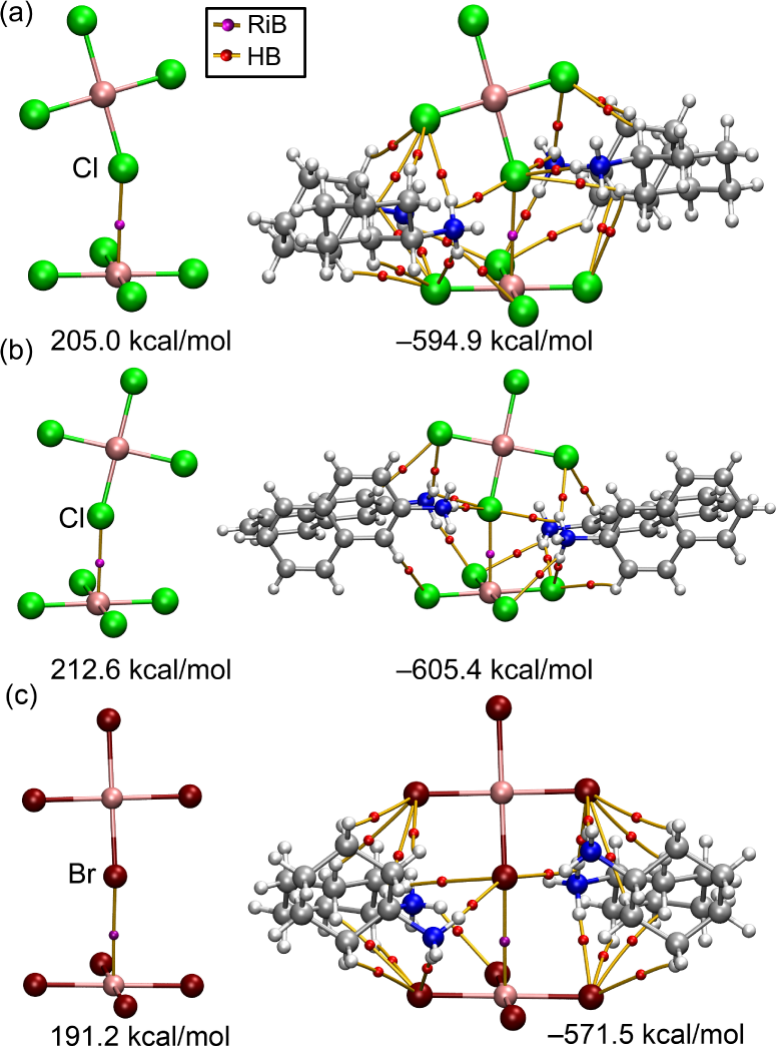
QTAIM analysis of the anion···anion dimeric (left) and neutral hexameric (right) assemblies of **1a** (a), **1b** (b), and **2a** (c). The binding energies are also indicated.

The dimerization energies computed in the absence of counterions are large and positive, indicating that these interactions are unfavorable due to strong electrostatic repulsion between the anions. However, upon inclusion of counterions (specifically, four cations) in the calculations, the binding energies become significantly negative.

Additionally, QTAIM analysis reveals a complex network of interactions, characterized by the presence of numerous BCPs (red spheres) and bond paths, which interconnect both anions and cations. This intricate interaction network, which includes NH···X and CH···X HBs, is nicely consistent with crystallographic observations and highlights the crucial role of counterions in stabilizing the system and facilitating noncovalent interactions.

NBO analysis was performed to examine the Cu···X contacts, evaluating the electron-donor properties of the X atom and the electron-acceptor capacity of Cu. In hole interactions, an antibonding orbital of the electrophilic atom interacts with a lone pair (LP) or a π orbital of the nucleophilic atom. The NBO analysis for the anion···anion dimers of **1a**, **1b**, **2a**, and the (4,4)-anion network (**2e** and **2f**) is presented in Figure 8. In all investigated systems, electron donation from an LP­(X) to a σ*­(Cu—X) antibonding orbital is observed, with stabilization energies of 0.5 kcal/mol for the anion···anion dimer of **1a**, 1.2 kcal/mol for **1b**, 0.3 kcal/mol for **2a**, and 1.0 kcal/mol for the dimer extracted from the (4,4)-anion network. Although the stabilization energies are relatively modest, these results support the existence and attractive nature of the Cu···X regium bonds. Notably, the higher stabilization energy observed in **1b** correlates well with the short experimental Cu···Cl distances in the (4,4)-anion network, emphasizing the important role of geometric factors in enhancing the stability of regium bonds in these systems.

**Figure 8:**
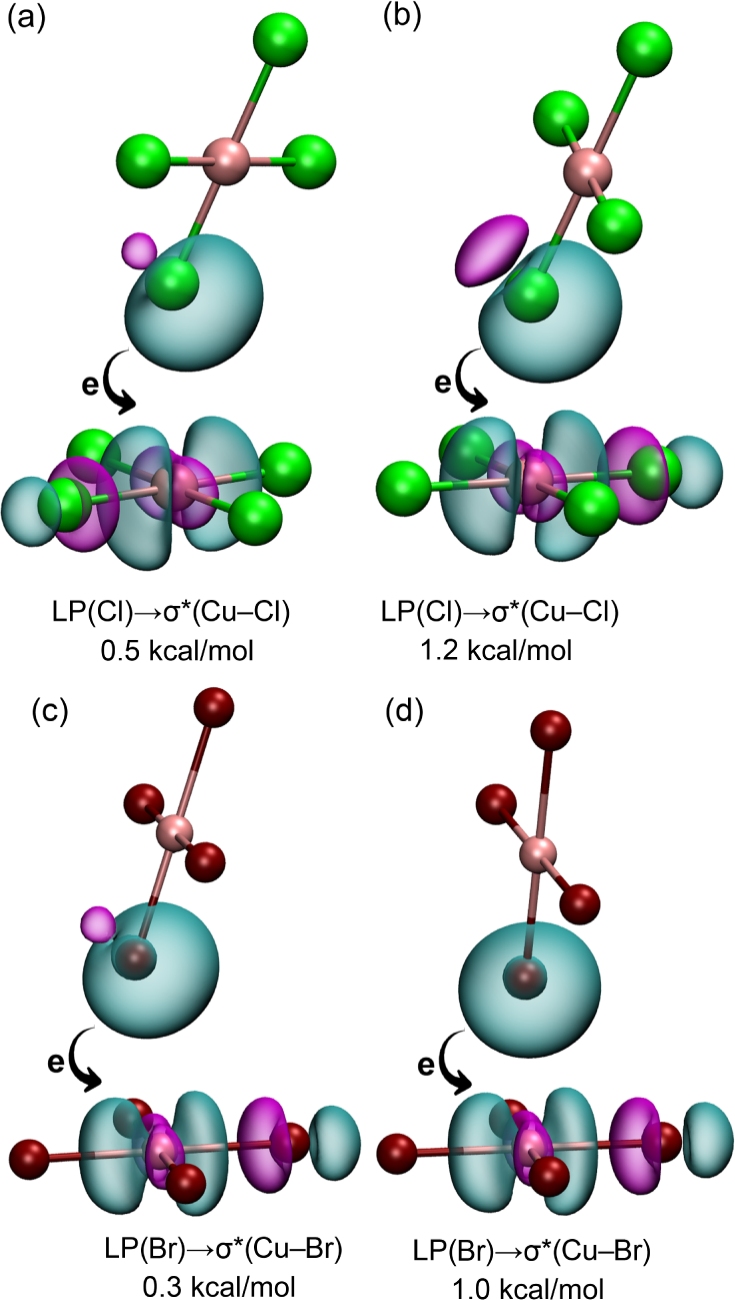
NBOs of the anion···anion dimers of **1a** (a), **1b** (b), **2a** (c), and (4,4)-anion net (d). The second order stabilization energies are also indicated.

Further investigation into the attractive nature of the Cu···X contacts in all compounds and their donor–acceptor dynamics was conducted using combined 2D plots of the Laplacian of electron density (∇^2^ρ) overlaid with 2D Reduced Density Gradient (RDG) maps, as illustrated in Figures 9 and 10. The ∇^2^ρ 2D plots provide insights into the covalent or noncovalent nature of the interactions, while the RDG maps effectively identify regions of noncovalent interactions, making these combined analyses essential for a comprehensive understanding of the bonding characteristics. Additionally, the sign of the second eigenvalue of the Hessian matrix of ∇^2^ρ (λ_2_) within these low RDG regions indicates the presence of attractive forces, further confirming the nature of the bond. To complement these findings, the Electron Localization Function (ELF) 2D map was utilized to delineate the nucleophilic and electrophilic regions within the anion···anion homodimers.

**Figure 9:**
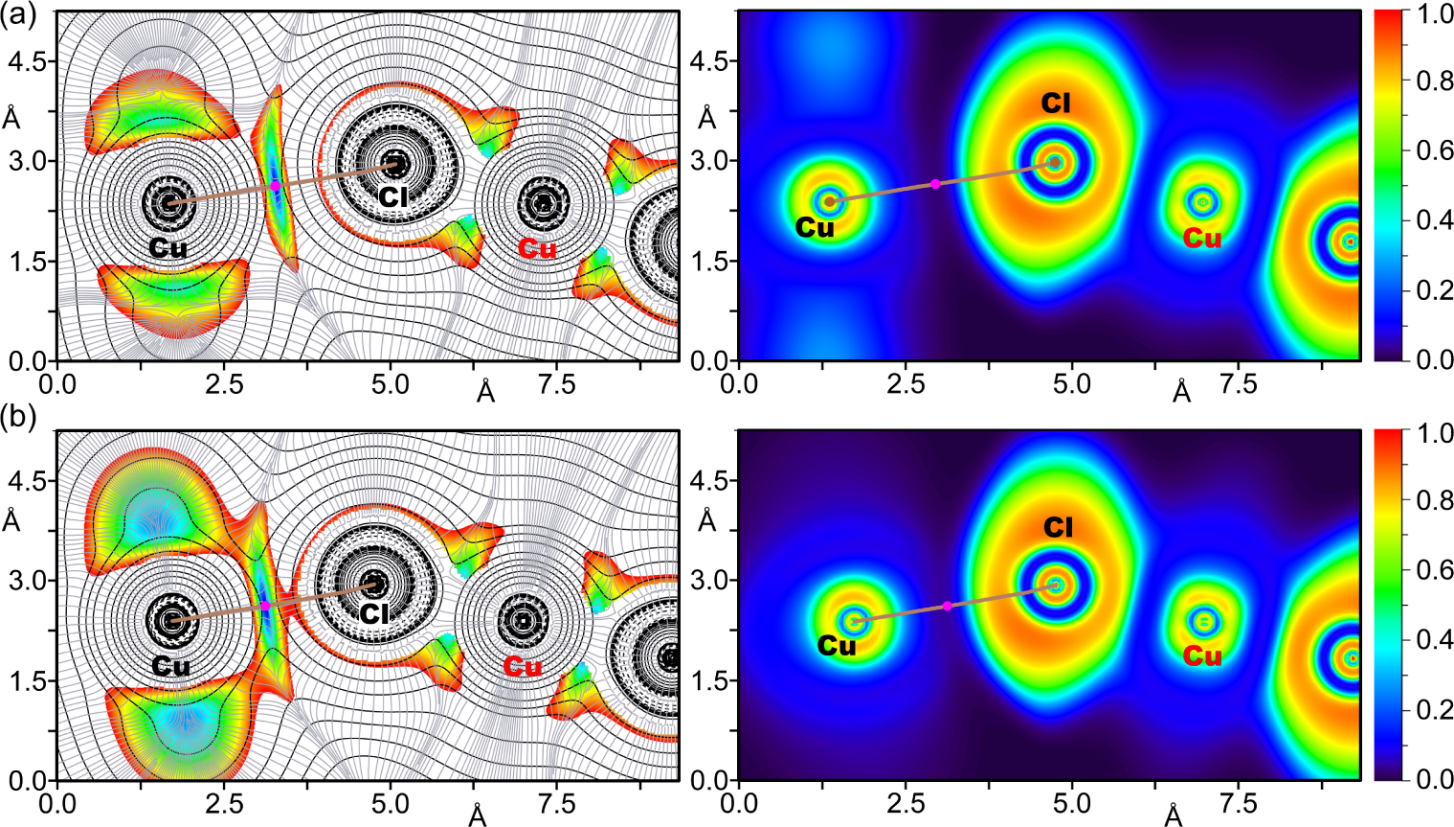
Left: 2D plot of the Laplacian (dashed lines for negative values and solid lines for positive ones) including the gradient lines (in gray) overlapped with the 2D RDG map for compounds **1a** (a) and **1b** (b). Right: ELF 2D maps of compounds **1a** (a) and **1b** (b) are represented in the plane defined by Cu···Cl—Cu atoms. The bond paths are represented as brown lines and BCPs as magenta dots. The RDG density cutoff is 0.05 au.

**Figure 10:**
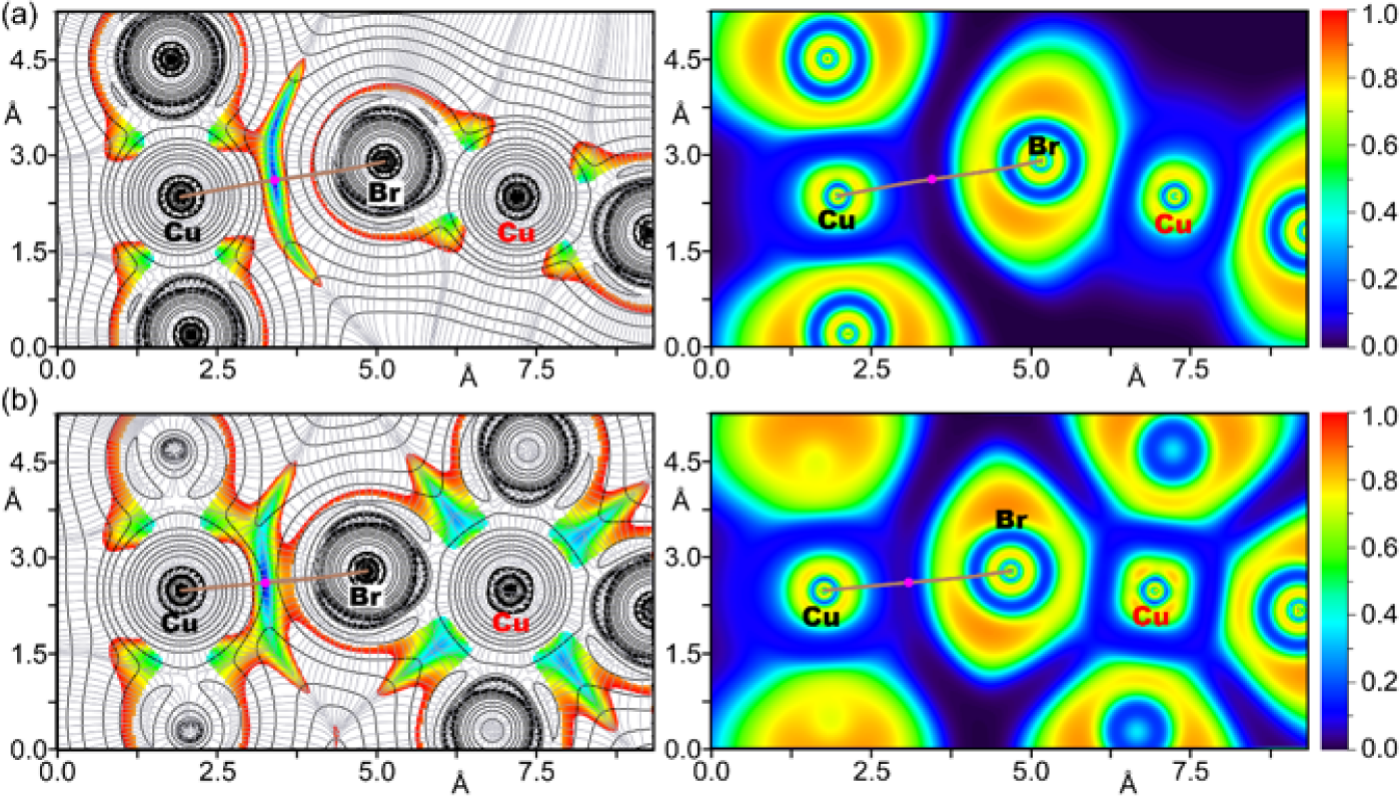
Left: 2D plot of the Laplacian (dashed lines for negative values and solid lines for positive ones) including the gradient lines (in gray) overlapped with the 2D RDG map for compounds **2a** (a) and **2b** (b). Right: ELF 2D maps of compounds **2a** (a) and **2b** (b) are represented in the plane defined by Cu···Br–Cu atoms. The bond paths are represented as brown lines and BCPs as magenta dots. The RDG density cutoff is 0.05 au.

This analysis, detailed in Figures 9 and 10, is supplemented by the bond critical point (BCP) parameters compiled in Table S8. Together, these analyses offer valuable insights into the electronic nature and stability of the Cu···X regium bonds in these complexes.

The 2D ∇^2^ρ analysis displays positive values (represented by solid line isocontours, Figure 9) between the Cl and Cu atoms in **1a** and **1b**, distinguishing between coordination bonds (Cu in red) and regium bonds (Cu in black). The same distinction is observed in complexes **2a** and **2b** (Figure 10).

This distinction is further clarified by the 2D-RDG maps, which show blue isocontours specifically in areas corresponding to elongated Cu···Cl/Br distances, effectively differentiating coordination bonds from regium bonds. The BCPs and bond paths that denote regium bonds are clearly marked on the 2D maps in Figures 9 and 10, where RDG values are near zero. The ELF 2D map provides additional insight into the contrasting characteristics of the Cu—Cl/Br coordination and regium bonds. It reveals a peak in ELF at the negative belt around the Cl and Br atoms (indicating lone pairs) and highlights the electrophilic nature of the Cu atoms. Furthermore, regions between Cl/Br and Cu atoms connected by coordination bonds are depicted in blue (ELF ≈ 0.4), suggesting some degree of electron localization indicative of electron sharing. In contrast, regions associated with regium bonds, marked by BCPs (magenta dots) in areas of minimal electron density (ELF ≈ 0.0, depicted in black), underscore the typical characteristics of noncovalent interactions.

It is important to note that the RiBs observed in these systems are intrinsically weak, particularly due to the anionic nature of both interacting species. The electron density values at the BCPs and the E^(2)^ energies obtained from NBO analysis are relatively small, generally below 0.01 a.u. and 1.2 kcal/mol, respectively. These modest values are consistent with the relatively long Cu···Cl/Br distances, which reduce orbital overlap and limit electron density accumulation. As such, while RiBs are clearly supported by structural and topological evidence, their energetic significance should be interpreted with appropriate caution.

## Conclusions

The results reported here demonstrate that adjacent CuCl_4_^2–^ and CuBr_4_^2–^ dianions can interact with each other via Cu···Cl/Br bondings that allow for the formation of supramolecular (4,4) networks in the solid. Theoretical studies on some of the examined salts support the noncovalent nature of these interactions and their regium bond character.

A detailed DFT analysis was conducted to investigate the electrostatic and electronic properties of Cu···X interactions within [CuX_4_]^2–^-based anion dimers (X = Cl, Br), using MEP surface analysis, QTAIM, and NBO approaches. The findings highlight the nature of these interactions, distinguishing coordination and regium bonds through MEP, RDG, and ELF mapping. Despite the electrostatic repulsive forces resulting from the coupling of [CuX_4_]^2–^ units, systems in which anion···anion dimers are paired with counterions are stable.

The reported results confirm the working hypothesis that the cation nature plays a pivotal role in activating the RiB donor ability of CuCl_4_^2–^ and CuBr_4_^2–^ anions. In all hybrid organic–inorganic systems discussed here, the cations are the protonated forms of primary amines (either aliphatic or aromatic). The N^+^H_3_ moieties form tight networks of short (and expectedly strong) NH···Cl/Br HBs that dissipate the negative charge of the dianions, increase the surface electrostatic potential at copper, and switch on their ability to form Cu···Cl/Br RiBs between different anionic units. Similar interactions are present in CuCl_4_^2–^ and CuBr_4_^2–^ salts reported in the CSD wherein cations are protonated primary amines, but not quaternary ammonium compounds.

A direct comparison between tetrachloridocuprate and tetrabromidocuprate anions reveals the significant role of the halogen atom in the anion···anion interactions. Chlorine, being smaller, less polarizable, and more electronegative than bromine, tends to form stronger and more stable bonds with copper.^62^ In contrast, bromine (larger, more polarizable, and less electronegative) forms weaker bonds with copper, making nontrivial the preparation of salts containing the CuBr_4_^2–^ anion. However, the major charge dissipation ensured by bromine favors the formation of deeper holes on copper, resulting in slightly stronger RiB interactions in the case of CuBr_4_^2–^.

These findings not only extend the concept of regium bonding to CuX_4_^2–^ anions but also give new insights about the factors allowing for the use of this interaction as a proper supramolecular tool for preparing functional materials such as semiconductors or organic–inorganic hybrid perovskites (OIHPs)^63^ with relevance in energy storage^64^ as well as in thermochromic,^65^ thermoelectric,^66^ ferromagnetic,^67^ and optoelectronic^68^ materials. Importantly, the findings reported here may enable the use of copper-based anionic species as valid and cost-effective alternatives to gold-based anions in advanced materials development.^35^

## Supplementary Material



## Notes

Synthesis and characterization of compounds studied in this work, CSD searches, additional structural figures, and theoretical methods data (PDF)
