# Supplementary material for: When CuCl4 2– and CuBr4 2– Form Anion···Anion Networks Assembled via Cu···Cl/Br Regium Bonds
Source: Cryst Growth Des. 2025 Jun 5;25(12):4338–47. doi: 10.1021/acs.cgd.5c00238 (PMC12186693; doi:10.1021/acs.cgd.5c00238)
Supplement: Supplementary file 1 [file cg5c00238_si_001.pdf]

Supporting Information

When $CuCl_4^{2-}$ and $CuBr_4^{2-}$ form anion···anion networks assembled via Cu···Cl/Br regium bonds

Cristina Lo Iacono,† Andrea Pizzi,† Kamran T. Mahmudov,‡ Rosa M. Gomila,§ Antonio Frontera,§ and Giuseppe Resnati*†

† NFMLab, Department Chemistry, Materials, Chemical Engineering "Giulio Natta"; Politecnico di Milano; 20133 Milano, Italy.

‡ Excellence Center, Baku State University, AZ 1148 Baku, Azerbaijan; Western Caspian University, AZ 1001 Baku, Azerbaijan.

§ Department of Chemistry, Universitat de les Illes Balears; Crta. de Valldemossa, 07122 Palma de Mallorca, Baleares, Spain

S1. Synthesis and Characterization of salts 1 and 2…………………2

S2. Cambridge Structural Database searches ………………………..5

S3. Crystallographic Details……………………………………………9

S4. Theoretical Methods………………………………………………18

S1. Synthesis and Characterization of salts 1 and 2

S1.1. Chemicals and materials

All starting materials and reagents were purchased from commercial suppliers (Merck and abcr) and used without further purification.

S1.2. Synthetic Methods

Adducts **1a-d**: A methanol solutions of $CuCl_2$ was added to aqueous HCl (6 N, 6 equivalents), the resulting mixture was added under stirring to an equimolar amount of the amine (cyclohexylamine for preparing **1a**, 2-naphthylamine for preparing **2b**, 1,12-dodecyldiamine for preparing **1c**, 1,6- hexyldiamine for preparing **1d**).
Adducts **2a,e,f**: aqueous HBr (6 N, 6 equivalents) was added to a methanol solution of $CuBr_2$; an equimolar amount of the amine dissolved in methanol (cyclohexylamine for preparing **2a**, isobutylamine for preparing **2e**, 1,4-diaminobutane for preparing **2f**) was added to the resulting mixture.
For both preparations, salts solutions were stirred at room temperature in a clear borosilicate vial for one hour. Slow isothermal evaporation of the solvents at room temperature afforded single crystals suitable for X-Ray diffraction after 3-5 days.

S1.3. Characterization

General Remarks

^1H and ^{13}C NMR spectra were recorded at ambient temperature on a Bruker AV-400 instrument and DMSO-d_6 was used as solvent. All the chemical shifts (δ) are given in ppm.
FT-IR spectra were obtained using a Nicolet Nexus FT-IR spectrometer equipped with UATR unit.

Di-cyclohexylammonium tetrachloridocuprate (**1a**)
IR (selected bands, cm^{-1}): 3088, 2931, 2858, 1584 1565, 1486, 1445, 1385, 999.
^1H NMR (400 MHz, DMSO-d_6) δ: 7.79 (brs, NH_3, 3H), 2.95 (m, CH, 1H); 1.89 (m, 4H), 1.72 (m, 4H), 1.57 (m, 2H).
^{13}C NMR (100 MHz, DMSO-d_6) δ: 49.78 (C1), 30.92 (C2, C6), 25.02 (C4), 24.16 (C3, C5).

Di-2-naphthylammonium tetrachloridocuprate (**1b**)
IR (selected bands, cm^{-1}): 2899; 2567; 1490; 889; 853; 809.
^{13}C NMR (101 MHz, DMSO-d_6) δ: 133.22, 132.18, 130.25, 128.31, 127,86, 127.73, 127.05, 121.83.

1,12-Dodecanediammonium tetrachloridocuprate (**1c**)
IR (selected bands, cm^{-1}): 3117, 3085, 2931, 1584, 1565, 1486.
^1H NMR (400 MHz, DMSO-d_6) δ: 7.79 (s, $(NH_3)_2$, 6H), 2.75(m, 4H), 1.53 (m, 4H), 1.35-1.18 (m, 16H).
^{13}C NMR (101 MHz, DMSO-d_6) δ: 39.30 (C1, C12), 29.38, 29.32, 29.01, 27.45, 26.29.

1,6-Hexanediammonium tetrachloridocuprate (**1d**)
IR (selected bands, cm^{-1}): 3100; 2931; 2862; 1565; 1482; 1438; 1216; 1106; 995; 985; 914.
^1H NMR (400 MHz, DMSO-d_6) δ: 7.72 (s, $(NH_3)_2$, 6H), 2.74 (m, $(CH_2N)_2$, 4H), 1.51 (m, $(CH_2CH_2N)_2$, 4H), 1.28 (m, $(CH_2CH_2\ CH_2N)_2$, 4H).
^{13}C NMR (101 MHz, DMSO-d_6) δ: 39.23(C1, C6); 27.23(C2, C5); 25.81(C3, C4).

Di-cyclohexylammonium tetrabromidocuprate (**2a**)
IR (selected bands, cm^{-1}): 3050, 2937, 2851, 1590, 1477, 1388, 1001.
^1H NMR (400 MHz, DMSO-d_6) δ: 7.77 (brs, NH_3, 3H), 2.96 (m, CH, 1H); 1.89 (m, 4H), 1.71 (m, 4H), 1.56 (m, 2H).
7.79 (brs, NH_3, 3H), 2.95 (m, CH, 1H); 1.89 (m, 4H), 1.72 (m, 4H), 1.57 (m, 2H).

^{13}C NMR (101 MHz, DMSO-d_6) δ: 49.80 (C1), 30.79 (C2, C6), 25.00 (C4), 24.17 (C3, C5).

Di-isobutylammonium tetrabromidocuprate (**2e**)
IR (selected bands, cm^{-1}): 3120; 2956; 2929, 1571; 1484; 1463; 1398; 1150; 1037; 997.
^{1}H NMR (400 MHz, DMSO-d_6) δ: 7.74 (brs, NH_3, 3H), 2.60 (d, CH_2N, 2H), 1.83(m, CH, 1H), 0.89 (d, $(CH_3)_2$, 6H).
^{13}C NMR (101 MHz, DMSO-d_6) δ: 46.24 (C–N), 26.83 (CH), 20.24 (CH_3).

1,4-Butanediammonium tetrabromidocuprate (**2f**)
IR (selected bands, cm^{-1}): 3143, 2925, 1582, 1475, 1437, 857.
^{1}H NMR (400 MHz, DMSO-d_6) δ: 7.79 (brs, $(NH_3)_2$, 6H), 2.79 (m, $(CH_2C\mathit{H_2}N)_2$, 4H), 1.58 (m, $(C\mathit{H_2}CH_2N)_2$, 4H).
^{13}C NMR (101 MHz, DMSO-d_6) δ: 39.13 (C1, C4), 24.90 (C2, C3).

S2. Cambridge Structural Database searches (Conquest version 2024.1.0)

Refcodes of the 612 structures containing isolated $CuCl_4^{2-}$ anion units (search criteria: tetravalent copper bonded to four monovalent chlorine atoms, two of them having a 0 charge, two of them having a -1 charge): COGYAC, DOJWOR02, EROWIU01, GEPKIY01, HAWWAH01, NEFWIE01, NITCOM, NODPEF, SIVMAP, SIWWUU, ABEYAK, ABEYAK01, ABEYIS, ABEYIS01, ABORUI, ACATIH, AGAWIO01, AGEVOY, AGOHIN, AHARUW, AHUWAB, AHUWEF, AHUWEF01, AHUWEF02, AHUWEF03, AHUWOP, ALIFEG, ALIFIK, AMUDCU10, ARUTUF, ASENAO, ASOXAH, AXACAF, AXADUY, AYALES, AYALIW, BAGLOK, BATGIP, BATGOV, BECSIN, BEGTOZ, BEHXIV, BEHXIV01, BEHXIV02, BEJKUZ, BEJKUZ01, BEJKUZ02, BEJKUZ03, BEQXUU, BEQXUU01, BEQYEF, BEQYEF01, BERPAT, BERPEX, BEZVIN, BEZVIN01, BICPEK, BICPIO, BIHCIE10, BINBOT, BOLTUU, BOMHER, BONZAG, BUCDAG, BUCDAG01, BUCDEK, BUCDOU, BUCQUO, BUCQUO01, BUDJES, BUGGUI, BUGGUI01, BUPSEL, CABKUP, CAJNAE, CAJNEI, CANVOD, CANVOD01, CANVOD02, CANVOD03, CATLOA, CATMUF, CATMUF01, CATMUF02, CEBMIG, CECDUK, CEFGEC, CEFJIJ, CEFSIS, CIXGAU, CIZWIT, COCNIS, CQAZCU, DADWEL, DAWQAW, DAWQEA, DERYAD, DIBVUG, DIBVUG01, DIBWOB, DIBWOB01, DIBWOB02, DIBWOB03, DIBWOB04, DMAMCU12, DOBCUT, DOJWOR, DOJWOR01, DUBHAL, DUDFUG, DUKCUJ, DUKNUT, DUKNUT01, DUKREI, DUKRIM, EAMCUC02, EBIYAS, EBIYIA, EBUNIA, EBUNOG, EBUNUM, EBUPAU, EGOFEL, EKAGON, EMAYOH, EPAXUP, EQAYUS, ERIYAI, EROWIU, ESAMOD, ESAPOG, ESAPOG01, ESUYEX, ETOYUI, ETUQOA, EVECAL, EVESUV, EVESUV01, EWEBUF, EWINOQ, EWOCEY, EZIWAL, FABCAP, FACFEU04, FAHNUB, FANZON, FASROI, FEBGOL, FEBHAY, FEGYEZ, FEJQUI, FIKFIQ, FIPBEL, FIPBEL01, FIQBUE, FISJEW, FISJEW01, FIVPUY, FOBSOG, FOBTAT, FOBTIB, FUDKEX, FUDKEX01, FUDKEX02, FUDLAU, FUHLOM, FUHLOM01, FUTRER, FUTRUH, GEMMIU01, GEMMIU02, GEPKIY, GICFEF, GIVZOB, GIWBOG, GIWYOD, GIZROX, GOTHOP, GOTLOT, GOWBUR, GUHWOX, GUTRIW, HAKXAW, HANSUO, HAWWAH, HEDSEO, HEPQIC, HEPQIC01, HEPWOO, HIBSAN, HIHBOP, HIHBOP01, HIPYAJ, HOKVAG, HOSSEO, HUMDUP, HUPVIX01, HUPVIX02, HUPVIX03, HUPVIX04, IDAPIM, IHOHEV, IHOTAB, IKINIC, IKINIC01, ILEDOT, INAXUS, INAXUS01, INITOR, IPRACU01, IRETOO, ITEYEM, ITEYEM01, IVOHEI, IVOHEI01, IVOHEI02, IWUNOE, IZEWEQ, JAJGEJ, JARCEL, JARCEL01, JARCIP, JEKFAK, JEKFEO, JEPKUK, JEPKUK01, JESBAO, JESBAO01, JIJYIO, JIRRUB, JIXPOW, JURJOX, KANZAC, KANZEG, KARHUJ, KAZDAQ, KELCEL, KIBKAK, KISLUU, KISLUU01, KOHLEB, KOHLEB01, KUHFAW, KUNJAE, KUNJAE01, KURPAP01, LADTER, LAMJEO, LAMJEO01, LAMJEO02, LAMSAV, LAMSAV01, LEJVUS, LEPFOE, LOKFOJ, LOSMEO, LUKQEQ, LURTAT, LURTAT01, LURTAT02, MACQEQ, MADPAK, MADPAK01, MAJLIT, MAMXUT, MAPJUJ, MAPJUJ01, MAPJUJ02, MASCUI, MATMOL, MATMUR, MBAMCC01, MBAMCC10, MEKCOV, MINQIJ, MINQOP, MINQUV, MOPRAK01, MOWYAZ, MOWYAZ01, MOWYAZ02, MPEACU12, MUGWUI, MXPEAD01, NACCUQ, NAHFAH, NAMNUN, NAMXUV, NANFUE, NAPFIW, NEZKAG, NIBKIU, NIJDAP, NIJZAH, NIKHUO, NIKHUO01, NIKQUV, NOCYEM, NOCYEM01, NOGLIG, NOGLIG01, NOGLIG02, NOGLIG03, NOGLIG04, NOGXOX, NOJYAM, NOWHEP, NOWHEP01, NUCLAB, NUTDOV. NUTDUB, NUTDUB01, OCERUM, OCIXII, OFOWIR, OFUYOG, OGAYII, OGAYII01, OGEMEV, OGEMEV01, OGINUP, OHELUJ, OHIWAE, OHIWIM, OHUDAX, OJUQIW, OLENEA, OLIHEZ, OSASIM, OTAQEH, OTOWEB, OWAZIY, OWEJOQ, OYAQOX, OYAQOX01, OYASIT, OYASIT01, OYEBAY, OYEBAY01, OYEBAY02, PAGVAY, PAGXOO, PALLUM, PAMTCC01, PAPXOX, PAQKUO, PAQKUO01, PAQLAV, PAQLAV01, PAQLEZ, PAQLOJ, PAQLOJ01, PATMON, PATMUT, PEBYAY, PEBYIG, PELGAP, PELGAP01, PEWNAJ, PEXBON, PEYCAA, PEYCII, PHPIPZ, PIMDUN, PIWXIG, PNCLCU01, PODTIM, POGGEY, POKRUF, POPJOW, POSQAR, POSYED, PRZCUA, PRZCUB, PUKZAZ, PUKZED, QAJBIO, QELVUB, QEMXAK, QEWXUM, QIHREG, QIHXAG, QISFAZ, QISFAZ01, QOLLEJ, QOLLEJ01, QOLSOB, QOTHUE, QOTHUE01, QOTHUE02, QQQFFP, QQQGAP, QQQGAS, QQQGAV, QUBHIF, RAHDOW,

RAVDOH, RAVFAV, RAVFEZ, RAVKAB, RAXNOV, REGVOP, RENCAP, RIFPUR, RIFPUR01, RISSOC, RIVLIU, RIVLOA, ROHWUI, ROVYOS, ROVYUY, ROVYUY01, ROZBIU, ROZDOC, RUBLUY, RUNXAC, RUPLAS, SADHOW01, SADHOW02, SADHOW03, SADHOW04, SADHOW05, SAGJUF01, SAGJUF02, SAJXIO, SAJXOU, SAJXUA, SAMDET, SAMDET01, SAMDET02, SARHUQ, SENDIC, SEZLEO01, SEZLIS01, SIKSOX, SINROY, SINROY01, SINROY02, SINROY03, SINROY04, SIXPEV, SOXLUM01, SOYDOA, SOYDOA01, SUPZOT, TAGSUS, TEZFEN, TEZFEN01, TEZFEN02, THAMCU01, THAMCU02, THAMCU10, TIBRUV, TIDCUI01, TIDDAP01, TIDGUM, TIDHIB, TIGCOD, TIJSEN, TIPVUN, TMECUC04, TOJHIK, TOJHIK01, TOJHIK02, TOJHIK03, TOJHIK04, TOJHIK05, TOJHIK06, TOJHIK07, TOJHIK08, TOJHIK09, TOJHIK10, TOJHIK11, TOJHIK12, TOJHIK13, TOJHIK14, TOJHIK15, TOJHIK16, TOJHIK17, TOJHIK18, TOJHIK19, TOLJOW, TUCROY, TUXPAG, UBEWIL, UBEYAF, UDAHIT, UFOYIA, UGURIX, UMUXOQ, UQAJUT, URASUD, URASUD01, USEHIM, UTUDIX, UZEKUH, VACGUB04, VACGUB05, VACGUB06, VECCEN, VEFREF, VEFRIJ, VEFZEL05, VEFZEL06, VEGLEB, VEGLIF, VEPCID, VEPKOR, VEPKOR01, VEPKOR02, VEPKOR03, VEPKOR04, VEPKOR05, VETMOX, VETNEO, VEWBOQ, VICFET, VICFET01, VICMIE, VIFWOX, VIYDEP, VOJMOY, VOJNOZ, VOJNUF, VOJPIV, VOJRIX, VORSIH, VORSON, WABMIX, WAKVUA, WATFUT, WAZJIS, WEBKUM, WEBPUR, WEBPUR01, WEBQAY, WEKKIK, WEMQAJ, WEMVAM, WEMVEQ, WEQKOV, WITXAB, WOCDEX, WOCDIB, WOKBIH, WOXJIF, WOXJIF01, WOXJIF02, WOXJIF03, WOXJIF04, WUTHOJ, WUTHOJ01, WUTHOJ02, WUTTUC, WUTVEO, WUTYIV, WUVFAW, XECMID, XEHKEB, XEQZOL, XEXDUD, XEYHOB, XEYVEC, XEYVEC01, XEYVEC02, XEYVEC03, XEYVEC04, XIGCID, XIGCOJ, XIGDAW, XINQAN, XINVOI, XOZNAC, XURHUQ, XURHUQ01, YAKYIT, YARTEQ, YECFEV, YEFVUD, YEFVUD01, YIFYIZ, YIHXIX, YOPNAS01, YOPNAS03, YOPNAS04, YUVXAR, ZAHRIN, ZAMCEV, ZENMEO, ZEPTEW, ZEXGUE, ZEYPUR. ZEYPUR01, ZICSOV, ZILTUN, ZILTUN01, ZILTUN02, ZIPHIP, ZISDOW, ZISDUC, ZISFAK, ZISHOA, ZISHUG, ZOHKEP, ZOZHII, ZOZHII01, ZOZHOO, ZUSYEU, ZZZPMY01, ZZZPMY05, ZZZPMY15, ZZZSLQ.

Refcodes of the 206 structures containing isolated $CuBr_4^{2-}$ anion units (search criteria: tetravalent copper bonded to four monovalent bromine atoms, two of them having a 0 charge, two of them having a -1 charge): HOGNIE, POCFIA, POCFOG, VIYBIS, YIZQEH, AGEVUE, AHUVUU, AHUWIJ, AHUWIJ01, AHUWIJ02, AHUWUV, AQAMOX, AYELUM, AYOJUU, BACHIX, BACHIX01, BACHOD, BACHUJ, BACJAR, BACJAR01, BOMHAN, BONZEK, BUDWIJ, BUDZOS, BUGTOP, BUGTOP01, BUVSIV, CACSOP, CANVUJ, CAWSUR, CEFFEB, CEFJOP, CUXWID, CUXWID01, DEPBOQ, DIBWAN, DIBWER, DIBWIV, DIBYET, DICZUO, DIRFAP, DIRFET, DOQHID, DUKSUY, DULXOY, DULXOY01, EMAYIB, EPALOX, EPEHAK, EPEHEO, EPEHIS, EPEHIS01, EPEHUE, ETUPAL, EYOSOC, EZIWEP, FENBOP, FISJIA, FIVKIH, FOBSIA, FOBSUM, FOBTEX, FORKUS, FUTTIX01, GEGBID, GEKCUW, GICFOP, GICFUV, GIVDEX, HANTAV, HEPDAH, HEPDAH01, HEPMAQ, HEPMAQ01, ICONAR, ICONEV, ICONIZ, ICONOF, IJACII, ILEDUZ, INEHER, ITUYUS, JEKDEM, JETDAQ, JIRROV, JOCYEG, JOLREJ01, JUKXES, JUKXES01, KAFHEG, KIJCIT, KIKNAX, KIKNAX01, KOYDIO, LAHYAU, LEJVOM, LEYNUB, LIDKEQ, LOBRUQ, LOBRUQ01, LUKQIU, LUYWAE, LUYWAE01, MEBXIE, MEKKEV, MODLIB, MOWXUS, NABNEK02, NABNEK03, NABNEK04, NACDAX, NANNID, NANNID01, NAZPAH, NAZPEL, NEWQUD, OCIXOO, OCUQUC, OFUNAH, OHIWEI, OHIWOS, OHUPAI, OKANAQ, OKANEU, OLIGEY, PAFYUR, PAXYET, PEBYEC, PEBYOM, PEXBUT, PIPQOW, PIPQOW01, POBJEZ, PUXQEE, PUXQEE01, QEWYEX, QINXAQ, QIPCEB, QIPCIF, QITTAQ, QITTEU, QOTJIU, QOTJIU01, QOTLAO, QQQCCD, QUBHOL, QUXTEJ, REGVIJ, ROKLUZ, RUFVOG, RUPLEW, SADHIQ01, SADHIQ02, SIGRIN, TABYIG, TABYOM, TABYOM01, TABYOM02, TABYOM03, TECSON, TEXDEG, TEXDEG01, TIFMEA, TIZZUY, UDEBEN, UDOYUH, UFAFEM, UFOYOG, UHAKUJ, UHAKUJ01, UHAKUJ02, ULAVAF, ULAVAF01, ULOWUP, UQUNEA, VICMEA, VULGEP, VULGIT, WALHIC, WEMTOY, WEMTUE, WEMVIU, WORHOD, WORHOD01, WUTHID, XAGLEW, XUGDOT, XUNZAK, XUNZAK01, XUNZAK02, XUNZAK03, XUNZAK04, XUNZAK05, XUNZAK06, XUNZAK07, XUNZAK08,

XUNZAK09, XURGAV, YACHIW, YATYUP, YATYUP01, YEJQUA, YIGMIO, YOBCUP, YOHRUL, ZEYQEC.

Refcodes of isolated $CuCl_4^{2-}$ anion units adopting a:
-- tetrahedral geometry (search criteria: tetravalent copper bonded to four monovalent chlorine atoms, two of them having a 0 charge, two of them having a -1 charge, Cl–Cu–Cl angles between 104° and 114°): NITCOM, BEQXUU, RIVLOA, TAGSUS, TIBRUV, XOZNAC;
-- seesaw geometry (search criteria: tetravalent copper bonded to four monovalent chlorine atoms, two of them having a -1 charge, one Cl–Cu–Cl angle between 170° and 180°, one Cl–Cu–Cl angle between 80° and 100°, four Cl–Cu–Cl angles between 87° and 93°): NIKHUO01;
-- square planar geometry (search criteria: tetravalent copper bonded to four monovalent chlorine atoms, two of them having a 0 charge, two of them having a -1 charge, two Cl–Cu–Cl angle between 170° and 180°, four Cl–Cu–Cl angle between 85° and 95°): EROWIU01, NEFWIE01, ABEYIS, ABEYIS01, AMUDCU10, ASOXAH, BEHXIV, BEHXIV0, BEHXIV02, BEJKUZ, BEJKUZ01, BEJKUZ02, BEJKUZ03, BEZVIN, BEZVIN01, BINBOT, BOPWUY, BORRUV, CEFJIJ, COCNIS, CRINCC, DUKREI, EAMCUC02, EDIACU20, EROWIU, ESAMOD, ESAPOG, ESAPOG01, EVECAL, EVESUV, FEGYEZ, FUDKEX, FUDKEX01, FUDKEX02, FUDLAU, FUHLOM, FUHLOM01, FUTRUH, GEMMIU, GEMMIU01, GEMMIU02, GIWYOD, HIPYAJ, IPRACU, IVOHEI, IVOHEI01, IVOHEI02, JEPKUK, JEPKUK01, JEPLEV, JOJREF01, KARHUJ, KIBKAK, KURPAP, KURPAP01, LAMJEO, LAMJEO01, LAMJEO02, MATMOL, MATMUR, METHCC10, MIXYEX, MPEACU10, MPEACU12, MUGWUI, NEFWIE, NIJDAP, NIKHUO, NOGLIG, NOGLIG01, NOGLIG02, NOGLIG03, NOGLIG04, NUTDOV, OCERUM, OGEMEV, OGEMEV01, OWEJOQ, OYAQOX, OYAQOX01, OYASIT, OYASIT01, OYEBAY, OYEBAY01, OYEBAY02, PAMTCC, PAMTCC01, PAQKUO, PAQKUO01, PAQLOJ, PAQLOJ01, PODTIM, POSQAR, POSYED, QOTHUE, QOTHUE01, QUFBAU, SAMDET, SAMDET01, SIDSON, UBEWIL, UBEYAF, URASUD, URASUD01, VACGUB04, VACGUB05, VACGUB06, VECCEN, VEFZEL05, VEFZEL06, VEPCID, VORSIH, VORSON. VUMDIR, WEBPUR, WEHVOU, WITXAB., WOKBIH, WUVDAT, WUVFAW, XIGCID, XIGCOJ, XIGDAW, XURHUQ01, YUVXAR, ZILTUN.

Refcodes of isolated $CuBr_4^{2-}$ anion units adopting a:
-- tetrahedral geometry (search criteria: tetravalent copper bonded to four monovalent chlorine atoms, two of them having a 0 charge, two of them having a -1 charge, Br–Cu–Br angles between 104° and 114°): DULXOY, JEKDEM, JUKXES, JUKXES01, KIKNAX, KIKNAX01, PIPQOW, QIPCIF, REGVIJ;
-- square planar geometry (search criteria: tetravalent copper bonded to four monovalent bromine atoms, two of them having a 0 charge, two of them having a -1 charge, two Br–Cu–Br angle between 170° and 180°, four Br–Cu–Br angle between 85° and 95°): EPEHIS, EPEHIS01, GEGBID, JEPLAR, JEPLIZ, NEFWOK, OLIGEY, QOTJIU, WIJWUH, XURGAV, YATYUP, YATYUP01.

Refcodes of the 78 structures wherein isolated $CuCl_4^{2-}$ anion units form a short Cu···Cl–Cu contact (search criteria: tetravalent copper bonded to four monovalent chlorine atoms, two of them having a 0 charge, two of them having a -1 charge, Cu···Cl contacts shorter than the sum of default van der Waals radii used by ConQuest): EROWIU01, NEFWIE01, SIWWUU, AMUDCU10, ARUTUF, BEHXIV, BEHXIV01, BEHXIV02, BEJKUZ, BEJKUZ01, BEJKUZ02, BEJKUZ03, BEZVIN, BEZVIN01, EAMCUC02, EDIACU20, EROWIU, ESAMOD, ESAPOG01, EVECAL, EVESUV, FEGYEZ, FUDKEX, FUDKEX01, FUDKEX02, FUDLAU, FUHLOM, FUHLOM01, IPRACU, IVOHEI, IVOHEI01, IVOHEI02, JEPKUK, JEPKUK01, JEPLEV, KARHUJ, KURPAP, KURPAP01, LAMJEO, LAMJEO01, LAMJEO02, MATMOL, MATMUR, NEFWIE, NIKHUO, NIKHUO01, NOGLIG, NOGLIG01, NOGLIG02, NOGLIG03, NOGLIG04, OWEJOQ, OYASIT, OYASIT01, OYEBAY, OYEBAY01, OYEBAY02, PAMTCC, PAMTCC01, SAGJUF, SAGJUF01, SAGJUF02, SAMDET, SAMDET01, UBEWIL, UBEYAF, URASUD,

URASUD01, VECCEN, VEFZEL05, VEFZEL06, VORSIH, VORSON, WEBPUR, WITXAB, WOCDIB, XURHUQ01, ZILTUN.

Refcodes of the 5 structures wherein isolated $CuBt_4^{2-}$ anion units form a short Cu···Br–Cu contact (search criteria: tetravalent copper bonded to four monovalent chlorine atoms, two of them having a 0 charge, two of them having a -1 charge, Cu···Br contacts shorter than the sum of default van der Waals radii used by ConQuest): CAYPOH, JEPLAR, JEPLIZ, NEFWOK, XURGAV.

S3. Crystallographic Details

Table S1. Crystal data and structure refinement for **1a.**

**Identification code**	**1a**
**Empirical formula**	$C_6H_{14}Cl_2Cu_{0.5}N$
**Formula weight**	202.85
**Temperature/K**	298.17(10)
**Crystal system**	orthorhombic
**Space group**	Pbca
**a/Å**	7.10420(10)
**b/Å**	8.60840(10)
**c/Å**	29.3721(5)
**α/°**	90
**β/°**	90
**γ/°**	90
**Volume/Å^3**	1796.27(4)
**Z**	8
**ρ_{calc}g/cm^3**	1.500
**μ/mm^1**	7.098
**F(000)**	844.0
**Crystal size/mm^3**	0.2 × 0.15 × 0.05
**Radiation**	Cu Kα (λ = 1.54184)
**2Θ range for data collection/°**	6.018 to 153.246
**Index ranges**	$-8 \le h \le 8$, $-10 \le k \le 9$, $-37 \le l \le 34$
**Reflections collected**	15344
**Independent reflections**	1818 [R_{int} = 0.0432, R_{sigma} = 0.0259]
**Data/restraints/parameters**	1818/0/89
**Goodness-of-fit on F^2**	1.055
**Final R indexes [I>=2σ (I)]**	R_1 = 0.0251, wR_2 = 0.0652
**Final R indexes [all data]**	R_1 = 0.0311, wR_2 = 0.0693
**Largest diff. peak/hole / e Å^{-3}**	0.28/-0.24
**CCDC Number**	2421519

Table S2. Crystal data and structure refinement for **1b.**

**Identification code**	**1b**
**Empirical formula**	$C_{10}H_{10}Cl_2Cu_{0.5}N$
**Formula weight**	246.86
**Temperature/K**	298.17(10)
**Crystal system**	monoclinic
**Space group**	$P2_1/c$
**a/Å**	18.7808(4)
**b/Å**	7.07203(14)
**c/Å**	7.81218(14)
**α/°**	90
**β/°**	92.4907(17)
**γ/°**	90
**Volume/Å^3**	1036.62(3)
**Z**	4
**ρ_{calc}g/cm^3**	1.582
**μ/mm^1**	6.287
**F(000)**	502.0
**Crystal size/mm^3**	0.2 × 0.2 × 0.05
**Radiation**	Cu Kα (λ = 1.54184)
**2Θ range for data collection/°**	9.428 to 153.394
**Index ranges**	$-23 \leq h \leq 22$, $-8 \leq k \leq 8$, $-9 \leq l \leq 9$
**Reflections collected**	18558
**Independent reflections**	2126 [R_{int} = 0.0709, R_{sigma} = 0.0343]
**Data/restraints/parameters**	2126/0/125
**Goodness-of-fit on F^2**	1.054
**Final R indexes [I>=2σ (I)]**	R_1 = 0.0359, wR_2 = 0.0974
**Final R indexes [all data]**	R_1 = 0.0437, wR_2 = 0.1033
**Largest diff. peak/hole / e Å^{-3}**	0.60/-0.40
**CCDC Number**	2421520

Table S3. Crystal data and structure refinement for **1c.**

**Identification code**	**1c**
**Empirical formula**	$C_{12}H_{30}Cl_4CuN_2$
**Formula weight**	407.72
**Temperature/K**	300.94(15)
**Crystal system**	triclinic
**Space group**	P-1
**a/Å**	7.20674(19)
**b/Å**	7.59326(19)
**c/Å**	16.9016(5)
**α/°**	92.893(2)
**β/°**	90.726(2)
**γ/°**	90.226(2)
**Volume/Å^3**	923.64(4)
**Z**	2
**ρ_{calc}g/cm^3**	1.466
**μ/mm^1**	6.902
**F(000)**	426.0
**Crystal size/mm^3**	0.2 × 0.2 × 0.05
**Radiation**	Cu Kα (λ = 1.54184)
**2Θ range for data collection/°**	10.482 to 153.08
**Index ranges**	$-8 \leq h \leq 8$, $-9 \leq k \leq 9$, $-21 \leq l \leq 20$
**Reflections collected**	17651
**Independent reflections**	3494 [R_{int} = 0.0533, R_{sigma} = 0.0393]
**Data/restraints/parameters**	3494/0/177
**Goodness-of-fit on F^2**	1.112
**Final R indexes [I>=2σ (I)]**	R_1 = 0.0625, wR_2 = 0.1679
**Final R indexes [all data]**	R_1 = 0.0695, wR_2 = 0.1726
**Largest diff. peak/hole / e Å^{-3}**	1.45/-0.90
**CCDC Number**	2421577

Table S4. Crystal data and structure refinement for **1d.**

**Identification code**	**1d**
**Empirical formula**	$C_4H_{12}Cl_{2.67}Cu_{0.67}N_{1.33}$
**Formula weight**	215.71
**Temperature/K**	99.99(10)
**Crystal system**	triclinic
**Space group**	P-1
**a/Å**	7.1388(2)
**b/Å**	7.6273(2)
**c/Å**	11.9927(7)
**α/°**	103.099(3)
**β/°**	91.833(4)
**γ/°**	90.040(2)
**Volume/Å^3**	635.66(5)
**Z**	3
**ρ_{calc}g/cm^3**	1.690
**μ/mm^1**	9.857
**F(000)**	330.0
**Crystal size/mm^3**	0.2 × 0.1 × 0.04
**Radiation**	Cu Kα (λ = 1.54184)
**2Θ range for data collection/°**	11.914 to 153.184
**Index ranges**	-8 ≤ h ≤ 9, -9 ≤ k ≤ 9, -14 ≤ l ≤ 14
**Reflections collected**	22453
**Independent reflections**	2452 [R_{int} = 0.0851, R_{sigma} = 0.0365]
**Data/restraints/parameters**	2452/0/115
**Goodness-of-fit on F^2**	1.075
**Final R indexes [I>=2σ (I)]**	R_1 = 0.1496, wR_2 = 0.4013
**Final R indexes [all data]**	R_1 = 0.1558, wR_2 = 0.4054
**Largest diff. peak/hole / e Å^{-3}**	3.38/-1.72
**CCDC Number**	2421578

Table S5. Crystal data and structure refinement for **2a.**

**Identification code**	**2a**
**Empirical formula**	$C_6H_{14}Br_2Cu_{0.5}N$
**Formula weight**	291.77
**Temperature/K**	100.00(10)
**Crystal system**	monoclinic
**Space group**	$P2_1/c$
**a/Å**	14.2589(2)
**b/Å**	7.51660(10)
**c/Å**	9.04940(10)
**α/°**	90
**β/°**	106.6480(10)
**γ/°**	90
**Volume/Å^3**	929.24(2)
**Z**	4
**ρ_{calc}g/cm^3**	2.086
**μ/mm^1**	11.647
**F(000)**	566.0
**Crystal size/mm^3**	0.15 × 0.1 × 0.05
**Radiation**	Cu Kα (λ = 1.54184)
**2Θ range for data collection/°**	6.47 to 152.64
**Index ranges**	$-17 \le h \le 17$, $-9 \le k \le 9$, $-10 \le l \le 11$
**Reflections collected**	16217
**Independent reflections**	1863 [R_{int} = 0.0320, R_{sigma} = 0.0140]
**Data/restraints/parameters**	1863/0/90
**Goodness-of-fit on F^2**	1.194
**Final R indexes [I>=2σ (I)]**	R_1 = 0.0199, wR_2 = 0.0537
**Final R indexes [all data]**	R_1 = 0.0204, wR_2 = 0.0539
**Largest diff. peak/hole / e Å^{-3}**	0.50/-0.49
**CCDC Number**	2422984

Table S6. Crystal data and structure refinement for **2e.**

**Identification code**	**2e**
**Empirical formula**	$C_{3.2}H_{9.6}Br_{1.6}Cu_{0.4}N_{0.8}$
**Formula weight**	212.59
**Temperature/K**	99.99(10)
**Crystal system**	monoclinic
**Space group**	$P2_1/c$
**a/Å**	14.0019(5)
**b/Å**	7.7312(3)
**c/Å**	7.7414(3)
**α/°**	90
**β/°**	103.354(3)
**γ/°**	90
**Volume/Å^3**	815.36(5)
**Z**	5
**ρ_{calc}g/cm^3**	2.165
**μ/mm^1**	13.185
**F(000)**	510.0
**Crystal size/mm^3**	0.21 × 0.15 × 0.05
**Radiation**	Cu Kα (λ = 1.54184)
**2Θ range for data collection/°**	6.488 to 153.476
**Index ranges**	$-17 \leq h \leq 17, 0 \leq k \leq 9, 0 \leq l \leq 9$
**Reflections collected**	1587
**Independent reflections**	1587 [R_{int} = ?, R_{sigma} = 0.0351]
**Data/restraints/parameters**	1587/0/64
**Goodness-of-fit on F^2**	1.129
**Final R indexes [I>=2σ (I)]**	R_1 = 0.1091, wR_2 = 0.2897
**Final R indexes [all data]**	R_1 = 0.1115, wR_2 = 0.2917
**Largest diff. peak/hole / e Å^{-3}**	3.94/-1.83
**CCDC Number**	2421579

Table S7. Crystal data and structure refinement for **2f.**

**Identification code**	**2f**
**Empirical formula**	$C_2H_7Br_2Cu_{0.5}N$
**Formula weight**	236.68
**Temperature/K**	100.02(10)
**Crystal system**	monoclinic
**Space group**	$P2_1/c$
**a/Å**	9.3808(3)
**b/Å**	7.8583(2)
**c/Å**	7.8677(2)
**α/°**	90
**β/°**	103.145(3)
**γ/°**	90
**Volume/Å^3**	564.79(3)
**Z**	4
**ρ_{calc}g/cm^3**	2.783
**μ/mm^1**	18.904
**F(000)**	442.0
**Crystal size/mm^3**	0.2 × 0.1 × 0.05
**Radiation**	Cu Kα (λ = 1.54184)
**2Θ range for data collection/°**	9.682 to 153.324
**Index ranges**	$-11 \le h \le 11$, $-9 \le k \le 9$, $-6 \le l \le 9$
**Reflections collected**	5369
**Independent reflections**	1130 [R_{int} = 0.0319, R_{sigma} = 0.0198]
**Data/restraints/parameters**	1130/0/53
**Goodness-of-fit on F^2**	1.162
**Final R indexes [I>=2σ (I)]**	R_1 = 0.0419, wR_2 = 0.1217
**Final R indexes [all data]**	R_1 = 0.0428, wR_2 = 0.1224
**Largest diff. peak/hole / e Å^{-3}**	2.15/-1.18
**CCDC Number**	2421582

Figure S1. Partial view of the crystal packing (ball and stick representation) of the tetrachlorido salt **1b** (along *b* axis) evidencing the layered architecture, the HBs (blue lines) at the layers interfaces, and the opposite orientation of cations in the organic layer resulting into an ABBA patter. Color code: whitish, hydrogen; grey, carbon; indigo, nitrogen; orange, copper; bright green, chlorine.

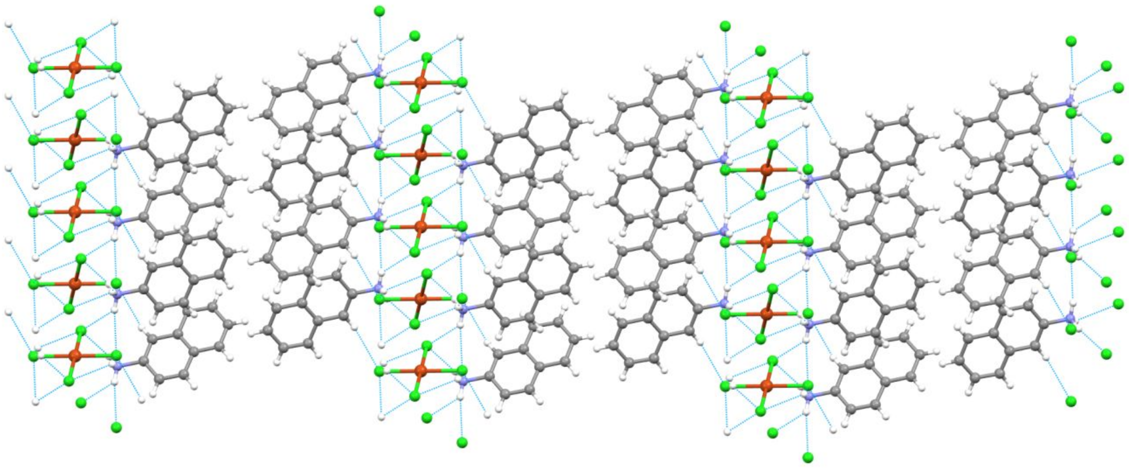

Figure S2. Partial view of the crystal packing (ball and stick representation) of the tetrachlorido salt **1d** (along *a* axis) evidencing the layered architecture, the HBs (blue lines) at the layers interfaces. Color code: whitish, hydrogen; grey, carbon; indigo, nitrogen; orange, copper; bright green, chlorine.

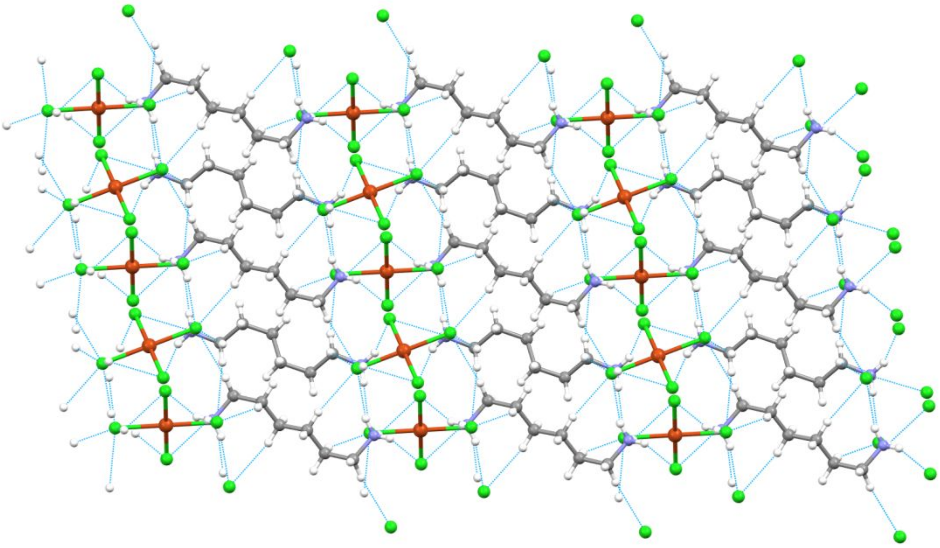

Figure S3. Partial view of the crystal packing (ball and stick representation) of the tetrabromiido salt **2a** (along *b* axis) evidencing the layered architecture, the HBs (blue lines) at the layers interfaces, and the opposite orientation of cations in the organic layer resulting into an ABBA patter. Color code: whitish, hydrogen; grey, carbon; indigo, nitrogen; orange, copper; light brown, bromine.

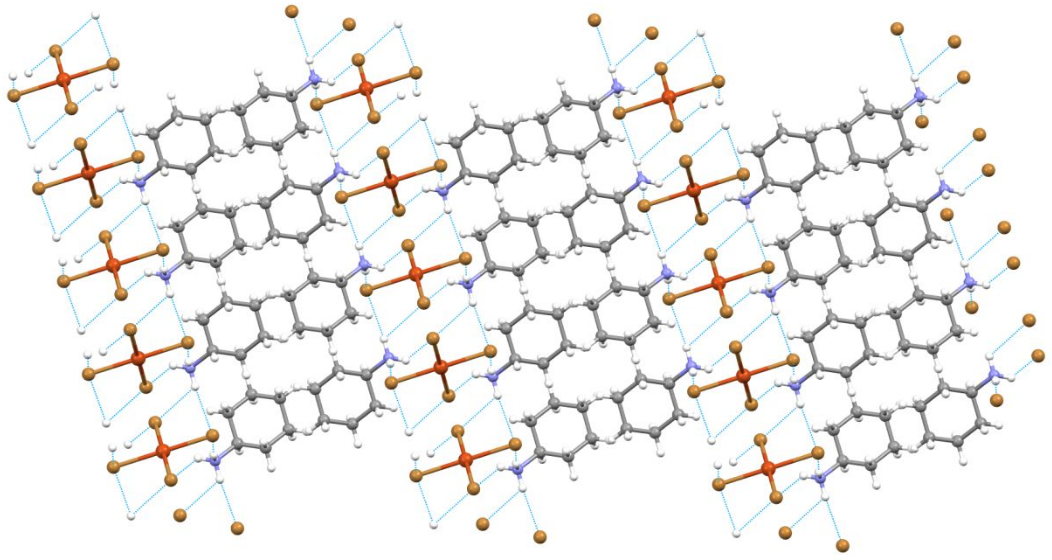

Figure S4. Partial view of the regium bonded (4,4) network (ball and stick representation) in the tetrachlorido salt **1a**. RiBs are black lines. Color code: orange, copper; bright green, chlorine.

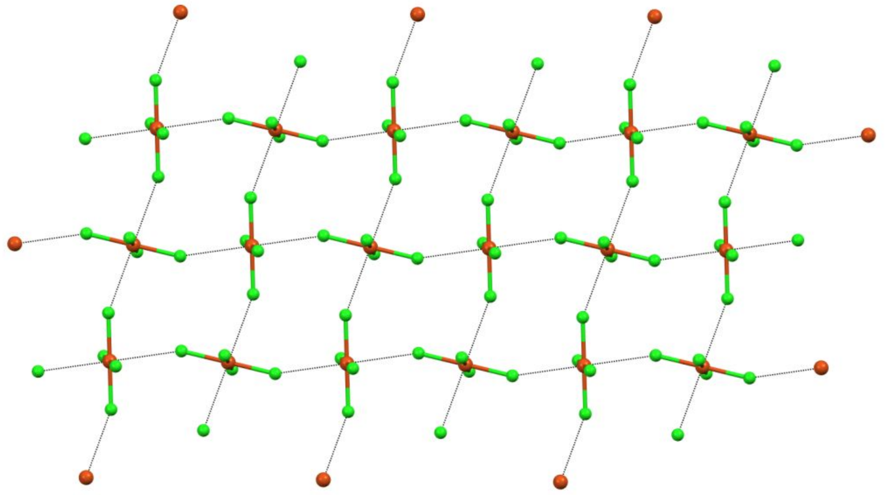

Figure S5. Partial view of the regium bonded (4,4) network (ball and stick representation) in the tetrachlorido salt **1d**. RiBs are black lines. Color code: orange, copper; bright green, chlorine.

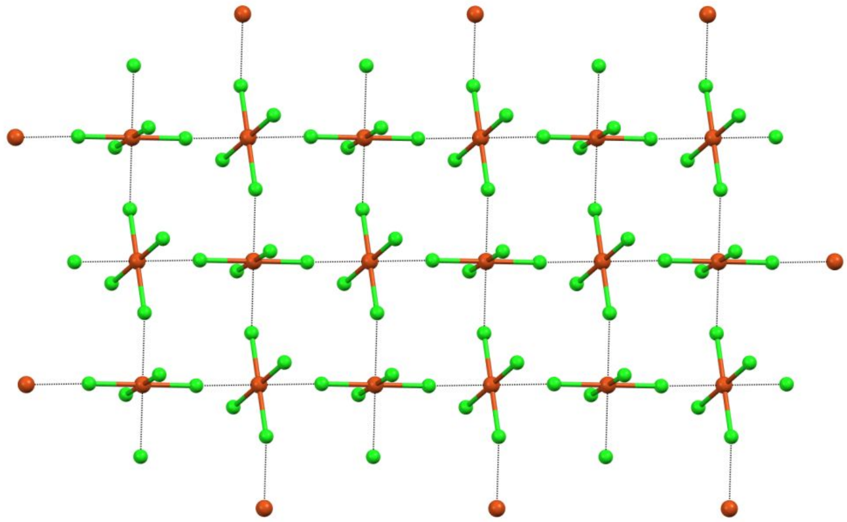

S4. Theoretical Methods

Table S8. QTAIM and ELF values in a.u. for the BCPs connecting the Cu atom to the Cl/Br atom that characterize the RgBs in compounds **1a**, 1b, 2a and 4,4- anion net.

**Dimer**	**BCP**	**ρ(r)**	**G(r)**	**V(r)**	**H(r)**	**$\nabla^2\rho(r)$**	**ELF**	**λ_2**
**1a**	Cu···Cl	0.0070	0.0042	–0.0040	0.0002	0.0174	0.0295	–0.0032
**1b**	Cu···Cl	0.0126	0.0086	–0.0083	0.0003	0.0354	0.0497	–0.0072
**2a**	Cu···Br	0.0058	0.0032	–0.0031	0.0001	0.0136	0.0262	–0.0023
(4,4) net	Cu···Br	0.0143	0.0091	–0.0092	–0.0001	0.0361	0.0647	–0.0078
